# Role of Satb1 and Satb2 Transcription Factors in the Glutamate Receptors Expression and Ca^2+^ Signaling in the Cortical Neurons In Vitro

**DOI:** 10.3390/ijms22115968

**Published:** 2021-05-31

**Authors:** Egor A. Turovsky, Maria V. Turovskaya, Evgeniya I. Fedotova, Alexey A. Babaev, Victor S. Tarabykin, Elena G. Varlamova

**Affiliations:** 1 Federal Research Center “Pushchino Scientific Center for Biological Research of the Russian Academy of Sciences”, Institute of Cell Biophysics of the Russian Academy of Sciences, 142290 Pushchino, Russia; m_turovskaya@mail.ru (M.V.T.); delf-fenka@rambler.ru (E.I.F.); 2 Institute of Neuroscience, Lobachevsky State University of Nizhniy Novgorod, 23 Prospekt Gagarina, 603950 Nizhny Novgorod, Russia; alexisbabaev@list.ru (A.A.B.); victor.tarabykin@charite.de (V.S.T.); 3 Institute of Cell Biology and Neurobiology, Charité-Universitätsmedizin Berlin, Berlin, Charitéplatz 1, 10117 Berlin, Germany

**Keywords:** Satb1, Satb2, calcium, glutamate receptors, GABA-receptors, gene deletion, neurons, cortex, signal transduction

## Abstract

Transcription factors Satb1 and Satb2 are involved in the processes of cortex development and maturation of neurons. Alterations in the expression of their target genes can lead to neurodegenerative processes. Molecular and cellular mechanisms of regulation of neurotransmission by these transcription factors remain poorly understood. In this study, we have shown that transcription factors Satb1 and Satb2 participate in the regulation of genes encoding the NMDA-, AMPA-, and KA- receptor subunits and the inhibitory GABA(A) receptor. Deletion of gene for either Satb1 or Satb2 homologous factors induces the expression of genes encoding the NMDA receptor subunits, thereby leading to higher amplitudes of Ca^2+^-signals in neurons derived from the Satb1-deficient (Satb1^fl/+^ * Nex^Cre/+^) and Satb1-null mice (Satb1^fl/fl^ * Nex^Cre/+^) in response to the selective agonist reducing the EC50 for the NMDA receptor. Simultaneously, there is an increase in the expression of the *Gria2* gene, encoding the AMPA receptor subunit, thus decreasing the Ca^2+^-signals of neurons in response to the treatment with a selective agonist (5-Fluorowillardiine (FW)). The Satb1 deletion increases the sensitivity of the KA receptor to the agonist (domoic acid), in the cortical neurons of the Satb1-deficient mice but decreases it in the Satb1-null mice. At the same time, the Satb2 deletion decreases Ca^2+^-signals and the sensitivity of the KA receptor to the agonist in neurons from the Satb1-null and the Satb1-deficient mice. The Satb1 deletion affects the development of the inhibitory system of neurotransmission resulting in the suppression of the neuron maturation process and switching the GABAergic responses from excitatory to inhibitory, while the Satb2 deletion has a similar effect only in the Satb1-null mice. We show that the Satb1 and Satb2 transcription factors are involved in the regulation of the transmission of excitatory signals and inhibition of the neuronal network in the cortical cell culture.

## 1. Introduction

Neuronal transcription factors regulate the expression of many receptors and intracellular signaling molecules involved in excitatory neurotransmission [1,2]. Great attention has been paid to transcription factors Satb1 and Satb2 in terms of their contribution to embryonic brain development. However, the effect of these factors on the Ca^2+^-signaling of neurons has not been practically investigated. The special AT-rich sequence-binding proteins 1 and 2 (Satb1/2) are nuclear matrix-associated proteins that exert multiple functions by influencing the structural organization of chromatin and interacting with several co-activators and co-repressors of transcription. Satb1 was first cloned from a cDNA library of the human cell line and found in thymocytes [3]. Further, this factor was discovered in other tissues and cells. *Satb1* ((MIM) 602075) gene resides on chromosome 3p24.3. The protein consists of 763 amino acids (~86 kDa molecular weight, migrates as 103 kDa on SDS gels) and includes six domains: nuclear localization signal, PDZ, BUR-binding domain, two Cut repeats (CUT1 and CUT2), and homeodomain (HD). Satb1 functions as a highly pleiotropic regulator of gene expression which dynamically alters the organization and epigenetic status of the chromatin [4]. Changes in Satb1 expression are associated with human tumors, including rectal cancer, cutaneous malignant melanoma, breast and prostate epithelia [5,6,7]. Satb1 is associated with highly metastatic breast cancer [4]. Most of the Satb1-regulated genes such as *errb2, abl1, mmp2, e-cadherin, vegfb, tgfb1*, and *kiss1* play an important role in the induction of carcinogenesis [8,9]. Satb1 via MAR-mediated interactions with DNA-regulatory elements is a transcriptional repressor [10]. Moreover, Satb1 may function as a “docking site” for several chromatin-modifier proteins that suppress gene expression [11]. Satb1 is more abundant in the brain and expressed exclusively in the neurons. Significant expression is observed in the neocortex, nucleus of the diagonal band, amygdala, and tegmental area. In contrast, in the case of the ventral midbrain, Satb1-positive neurons were observed only in the small part of the substantia nigra, ventral tegmental area, and retrorubral field, but no Satb1-positive neurons were found in the inferior colliculus. Thus, Satb1-positive neurons were predominantly dopaminergic [12]. The transcription factor Satb1 is extensively expressed in SST^+^-, CR^+^-, and NPY^+^-interneurons, while the expression was not observed in VIP^+^-interneurons [13,14]. Mice with Satb1 mutation are characterized by incomplete eye-opening and the clasping reflex [15].

The *Satb2* ((MIM) 608148) gene localizes in chromosome 2q32–q33, spans 191 kb, and contains 11 exons. Its open reading frame begins in exon 2, with the first stop codon in exon 11, predicting a 733 amino acid protein. The protein contains a Pfam-B_10016 domain required for dimerization (residues 57–231), two CUT domains (352–437 and 482–560), and a homeodomain (614–677) [16]. Satb2 is highly conserved (99.6%) among the members of the transcription-factor gene families which members bind to nuclear matrix attachment regions and appear to be involved in the regulation of the tissue-specific organization of chromatin [17,18]. Furthermore, Satb2 is a target for SUMOylation—a reversible modification of the protein that modulates its activity [18]. A single case of a de novo nonsense mutation in Satb2 has been described in individuals with cleft palate, osteoporosis, profound mental retardation, epilepsy, a jovial personality, and craniofacial dysmorphism including gum hyperplasia, mandibular hypoplasia, and anterior pointing incisors [19]. Satb2 is a genetic risk locus for schizophrenia and a regulator of several miRNAs responsible for the translation of proteins that control certain aspects of synaptic plasticity and memory formation: miR-124, miR-125b, miR-132, miR-212, miR-381, miR-326, and miR-19b [20,21]. Alterations in the expression and activity of Satb2 cause Satb2-associated syndrome in humans. SAS (OMIM 612313) is a clinically recognizable syndrome characterized by neurodevelopmental and behavioral abnormalities, palatal and dental anomalies, dysmorphic features, and frequent skeletal pathology [22,23,24]. Patients with SAS are characterized by deep mental retardation, and learning difficulties [22,25].

Both of these transcription factors are involved in the regulation of the growth and development of various types of brain neurons, both in the embryonic and postnatal periods [17]. We have previously shown that mutations of the Sip1 or MeCP2 transcription factor cause a disturbance of Ca^2+^-signaling in neurons and astrocytes [26,27].

Changes in the expression level and activity of glutamate receptors upon deletion of transcription factors Satb1 and Satb2 may be considered one of the key fundamental mechanisms that determine the triggering of the neural network to a hyperexcitation state. Despite a sufficient number of works devoted to the role of Satb1 and Satb2 in neurogenesis and brain development, there are no studies demonstrating the changes in intracellular calcium signaling when these transcription factors are impaired in neurons. In our paper, we found that the expression of Satb1 and Satb2 is necessary for the development of excitatory and inhibitory neurotransmission, and conditional knockout of these transcription factors induces alterations in the expression of glutamate receptors responsible for the excitation of neural cells and the inhibitory GABA(A) receptor. At the level of intracellular signaling, the sensitivity of glutamate and GABA receptors to selective agonist changes, and the differences in the amplitude of Ca^2+^-neuron signals are registered.

## 2. Materials and Methods

Experimental protocols were approved by the Bioethics Committee of the Institute of Cell Biophysics. Experiments were carried out according to Act708n (23 August 2010) of the Russian Federation National Ministry of Public Health, which states the rules of laboratory practice for the care and use of laboratory animals, and the Council Directive 2010/63 EU of the European Parliament on the protection of animals used for scientific purposes.

### 2.1. Animals

We used conditional knockout mice carrying Satb1^flox^ or Satb2^flox^ allele in which exon is flanked by loxP sites, and thereby can be excised by recombination with Cre.

A conditional Satb1 allele was generated via homologous recombination using a targeting construct which is described in detail in the work of Close et al. [28]. LoxP sites in a targeting construct were placed in nonconserved regions for coding exon 2 and exon 3. The Cre-mediated deletion of exons 2 and 3 results in premature termination of Satb1 mRNA translation, due to the creation of several in-frame stop codons. To obtain Satb1 conditional knockout, Satb1^flox^-mice were interbred with mice harboring Cre recombinase as a ‘knock-in’ into the NeuroD6 locus as a driver line for Cre-mediated recombination in cortical projection neurons starting at E12 [29]. The mouse colonies were delivered to the SPF animal facility at the Lobachevsky State University of Nizhni Novgorod from the Institute of Cell Biology and Neurobiology, Charité-Universitätsmedizin Berlin.

The Satb2 conditional allele, which can be inactivated by the expression of Cre-recombinase, was generated in R.G.’s laboratory [30]. Satb2^flox^-mice were taken to Charité-Universitätsmedizin from Max-Plank Institute for Experimental Medicine, Gottingen, Germany, and then, like Satb1^flox^, they were bred to NEX-Cre mice.

Mice were kept in SPF cages 40 cm × 25 cm × 15 cm under standard laboratory conditions: a 12 h light circuit, 22 °C. Animals had free access to food and water. For housing, individually ventilated GM500 cages manufactured by Tecniplast (Buguggiate, Italy) with a floor area of 501 cm^2^ were used. In the nests, the harem type of housing (2 females + 1 male) was carried out. After weaning, on the twenty-first–thirtieth postnatal day, the transplanting of the offspring into a separate cage and the taking of material for genotyping (tip of the tail) occurs. All animals were housing with littermates of the same sex in a group of 2–5 mice.

**Figure 1:**
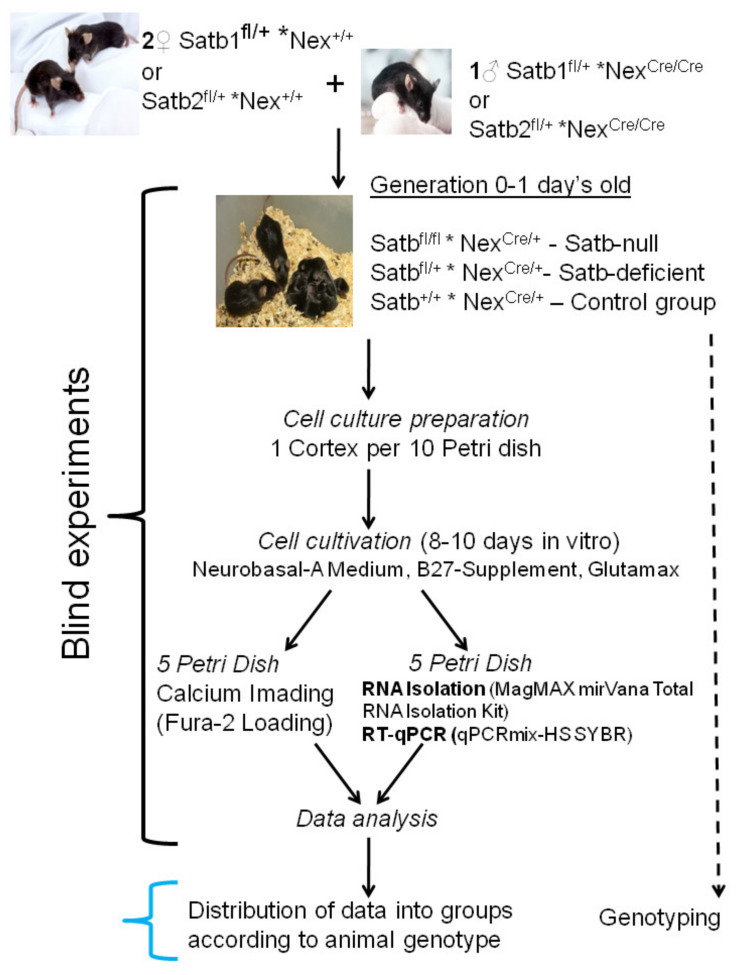
An experimental strategy. In the generation there was Satb-deficient (Satb^fl/+^ * Nex^Cre/+^), Satb-null mice (Satb^fl/fl^ * Nex^Cre/+^) and Control (Satb^+/+^ * Nex^Cre/+^) mice. The cerebral cortex from one mouse was used to obtain the cell culture. The cell suspension was inoculated at the rate of one cortex per 10 Petri dishes. The cells were cultured up to 8–10 days in vitro (DIV). Then five Petri dishes were used for [Ca^2+^]_i_ dynamics registration and total RNA was isolated from the other five Petri dishes and RT-qPCR was performed. This approach makes it possible to reliably compare neuroimaging data with changes in gene expression. Simultaneously with the data analysis, mice were genotyped, and then the results were divided into groups.

Newborn mice were used to obtain neuroglial cell cultures of the cerebral cortex. The tails of these mice were frozen and stored for subsequent genotyping. An experimental strategy is shown in Figure 1. In brief, cells were grown up to 8–10 days in vitro (DIV), loaded with the Fura-2 calcium-sensitive probe, and [Ca^2+^]_i_ dynamics were registered. At the stage of data analysis, genotyping was carried out, the distribution of experimental data in accordance with the genotype of animals, while at the previous stages, blinded experiments were carried out.

For genotyping, the tail cuts were dissolved in 0.3 mL of lysis buffer (100 mM Tris-HCl pH 8.5, 5 mM EDTA, 200 mM NaCl, 0.2% SDS, 100 μg/mL proteinase K) at 55 °C for 2–10 h. The non-lysed tissue was removed by centrifuging the samples for 10 min at 10,000 rpm. The DNA was precipitated by adding an equal volume of isopropanol, then mixed and centrifuged (15 min, 13,000 rpm). The precipitated DNA was washed twice in 80% ethanol, air-dried, and dissolved in 50 μL of sterile distilled water. All PCR reactions were carried out in a volume of 20 μL.

The following primers were used to determine the amplified product. Satb1-floxed allele and wild-type allele: 5′-GCATGTTTGTCTGTGTGCC-3′; 5′-CAGAAACAGTCTGGAGGGAGG-3 ′. Amplification program was as follows: 95 °C, 3 min; 95 °C, 30 s; 55 °C, 30 s; 72 °C, 30 s; 72 °C, 5 min, 30 cycles. The wild-type allele product is ∼120 bp, knockout ∼160 bp.

Satb2-floxed allele and wild-type allele: 5′-CAAGAGAGCCATCCAACTGC-3′; 5′-AACCATCAGGCTGCCTCAACC-3′; 5′-CCAGACCGCGCGCCTGAAGA-3′. Amplification program was as follows: 95 °C, 3 min; 95 °C, 10 s; 54 °C, 15 s; 72 °C, 30 s; 72 °C, 5 min, 33 cycles. The wild-type allele product is ∼200 bp, knockout ∼450 bp.

Primers for amplification of the NexCre allele and the wild-type allele: 5′-CCG CATAACCAGTGAAACAG-3′; 5′-GAGTCCTGGAATCAGTCTTTTC-3′; 5′-AGAATGTGGAGTAGGGTGAC-3′. Amplification program: 95 °C, 3 min; 95 °C, 20 s; 54 °C, 30 s; 72 °C, 60 s; 72 °C, 2 min, 35 cycles. The product of the wild-type allele is ∼750 bp, the NexCre allele is ∼500 bp.

### 2.2. Cell Culture Preparation

A total of 28 mice were used in the experiments. To obtain cell cultures, 8 control mice (Satb^+/+^ * Nex^Cre/+^), 6 Satb1-deficient (Satb1^fl/+^ * Nex^Cre/+^) and Satb2-deficient (Satb2^fl/+^ * Nex^Cre/+^) mice, 6 Satb1-null mice (Satb1^fl/fl^ * Nex^Cre/+^), and 8 Satb2-null mice (Satb2^fl/fl^ * Nex^Cre/+^) mice, respectively were used. Mixed neuroglial cell cultures were prepared as described in detail previously [31,32,33]. The cortex of one mouse was used to obtain ten Petri dishes with culture to avoid the variation in the gene expression and signaling system activity between individual mice. Briefly, 0–1 day-old pups were euthanized by halothane overdose and decapitated. The mouse cerebellar cortex was excised with clippers, put in a test tube, incubated for 2 min, and the supernatant was removed with a pipette. The cells were then covered with 2 mL trypsin (0.1% in Ca^2+^- and Mg^2+^-free Versene solution, SAFC Biosciences, Inc., Lenexa, KS, USA, Cat. #59427C) and incubated for 15 min at 37 °C under constant shaking at 600 rpm. Trypsin was then inactivated by an equal volume of cold embryo serum, and the preparation was centrifuged at 300 g for 5 min. The supernatant was discarded, and cells were washed twice with Neurobasal A medium (Thermo Fisher Scientific, Waltham, MA, USA, Cat. #10888022) before being resuspended in Neurobasal-A medium containing glutamine (0.5 mM, Sigma-Aldrich, St. Louis, MO, USA, Cat. #G7513), B-27 (2%, Thermo Fisher Scientific, RRID: CVCL_A315), and gentamicin (20 μg/mL, Sigma-Aldrich, Cat. #G1397). Moreover, 200 μL of the suspension was put in a glass ring (internal diameter of 6 mm) resting on a round 25 mm coverslip (VWR International, Radnor, PA, USA, Cat. #48382-085) which had been coated with Poly-L-lysine. The glass ring was removed after a 5 h incubation period in a CO_2_-incubator (37 °C) and the culture medium (2/3 of the volume) was replaced every 3 days. The density of plated cells was 15.000 cells/cm^2^ and the age of the neuronal cell culture was 8–10 days in vitro (DIV).

The use of 2D neuronal monoculture introduces some limitations in obtaining results and interpreting data, in contrast to 3D perfused cellular models [34,35], since, in addition to astrocytes, the brain contains endothelial cells, microglia, etc. However, the ratio of neurons to astrocytes in our cell cultures is in good agreement with the brain and the neuronal network differentiates during maturation. In addition, a simplified in vitro cellular model is required for titration experiments of receptor activity. In the cell cultures of the cerebral cortex for 10 DIV obtained from Satb1-deficient and Satb1-null-mice, the number of astrocytes averages 20.8 ± 8% and 22.3 ± 5%, and in the control group (Satb1^+/+^ * Nex^Cre/+^)—22 ± 8% (Supplementary, Figure S1A). Similarly, in cell cultures from mice with a Satb2 deletion, the percentage of astrocytes is 21 ± 7% for Satb2-deficient and 20 ± 4% for Satb1-null, and in the control (Satb2^+/+^ * Nex^Cre/+^)—24.2 ± 4% (Supplementary, Figure S1A). Thus, in the cell cultures obtained and grown under the conditions described above, most of the cells are neurons.

#### 2.2.1. Immunocytochemical Method

In order to detect Satb1, Satb2, GFAP, and NeuN in cells, we used an immunocytochemical assay. The cells were fixed with 4% paraformaldehyde + 0.25% glutaraldehyde in PBS for 20 min and washed three times with ice-cold PBS for 5 min. Glutaraldehyde was added into the fixative solution to minimize the washing of BDNF from cells during permeabilization. To permeabilize cells, we used 0.1% Triton X-100 solution for 15 min. Fixed cells were incubated in 10% donkey serum for 30 min at room temperature to block non-specific antibody binding sites. The cells were then incubated with primary antibodies against investigated proteins for 12 h at 4 °C. The fixed cells were subsequently washed with PBS (3 times for 5 min) and probed with secondary antibodies conjugated with a fluorescent label manual. We used purified mouse monoclonal anti-Satb1 antibody (BioLegend, RRID: AB_2565610, Cat. No. 676102), rabbit polyclonal antibody to GAD65+GAD67 (Thermo Fisher Scientific, RRID: AB_2263114), mouse monoclonal anti-Satb2 antibody (Abcam, Cambridge, UK, RRID: AB_882455), rabbit polyclonal Anti-NeuN antibody (Abcam, RRID: AB_10711153), rabbit polyclonal anti-NMDAR2B antibody (Invitrogen, Thermo Fisher Scientific, Waltham, MA, USA, Cat. No. PIPA585632), mouse monoclonal anti-NMDAR2A antibody (Abcam, ab240884), GluR1 polyclonal antibody (Thermo Fisher Scientific, Cat. No. PA5-95207), GluR2 monoclonal antibody (6C4) (Thermo Fisher Scientific, Cat. No. 32-0300), rabbit polyclonal anti-vesicular GABA transporter (VGAT) antibody (Merck Millipore, Burlington, MA, USA, Cat. No. AB5062P), purified mouse monoclonal anti-GFAP antibody (BioLegend, San Diego, CA, USA, RRID: AB_2632644), donkey polyclonal secondary antibody to rabbit IgG (H+L) (Alexa Fluor-647) (Jackson ImmunoResearch Europe LTD, Cambridge, UK, RRID: AB_2492288), donkey anti-rabbit IgG (H+L) highly cross-adsorbed secondary antibody (Alexa Fluor-488) (Thermo Fisher Scientific, Cat. No. A-21206), donkey polyclonal secondary antibody to mouse IgG-H&L (Alexa Fluor-594) (Abcam, RRID: AB_2732073), goat anti-mouse IgG (H+L) highly cross-adsorbed secondary antibody (Alexa Fluor-633) (Thermo Fisher Scientific, Cat. No. A-21052), and donkey polyclonal secondary antibody to rabbit IgG-H&L (Alexa Fluor-555) (Abcam, RRID: AB_2636997). Dilutions of primary and secondary antibodies were performed according to the manufacturer’s recommendations for immunocytochemical staining. The fluorescence of antibodies was visualized with an inverted confocal microscope Leica TCS SP5 (Leica, Hamburg, Germany). Registration of the secondary antibodies fluorescence for the control and experimental groups of cell cultures was carried out at the same microscope setting. Fluorescence analysis was performed in ImageJ 2002 software (RRID: SCR_003070, U. S. National Institutes of Health, Bethesda, MD, USA) using the analyze particles and time series analyzer plugins.

#### 2.2.2. Assessment of Cell Viability and Apoptosis

Cell death (apoptosis or necrosis processes) in the cell culture was assessed by simultaneous staining of cells with Propidium iodide (PI, 1 µM) and Hoechst 33,342 (HO342, 1 µM). It is known that viable cells are not permeable to PI, while Hoechst 33,342 penetrates through the plasma membrane, staining the chromatin. According to a commonly used method [31,36], cortical cells were defined as apoptotic if the intensity of Hoechst 33,342 fluorescence was 3–4 times higher compared to Hoechst 33,342 fluorescence in healthy cells, indicating chromatin condensation, which can occur as a result of apoptosis induction. The fluorescence of the probes was registered with a fluorescent system based on an inverted fluorescent microscope Axio Observer Z1 equipped with a high-speed monochrome CCD-camera Hamamatsu ORCA-Flash 2.8. The Lambda DG-4 Plus illuminator (Sutter Instruments, Novato, CA, USA) was used as a source of excitation. To excite and record fluorescence of the probes, we used: Filter Set 01 with excitation filter BP 365/12, beam splitter FT395, emission filter LP 397; Filter Set 20 with excitation filter BP 546/12, beam splitter FT560, and emission filter BP 575-640. Five different fields were analyzed for each coverslip. Each experiment was repeated three times with separate cultures.

### 2.3. Fluorescent Ca^2+^ Measurements

Experiments were carried out in the daytime. The measurements of [Ca^2+^]_i_ were performed by fluorescence microscopy using Fura-2/AM (Thermo Fisher Scientific, Cat. #F1221), a ratiometric fluorescence calcium indicator. Neurons were loaded with the probe dissolved in Hanks balanced salt solution (HBSS) composed of (mM): 156 NaCl, 3 KCl, 2 MgSO_4_, 1.25 KH_2_PO_4_, 2 CaCl_2_, 10 glucose, and 10 HEPES, pH 7.4, at a final concentration of 5 μM at 37 °C for 40 min with subsequent 15 min washout. Coverslip containing the cells loaded with Fura-2 was then mounted in the experimental chamber. To measure free cytosolic Ca^2+^ concentration, we used the Carl Zeiss Cell Observer and an inverted motorized microscope Axiovert 200M with a high-speed monochrome CCD-camera AxioCam HSm with a high-speed light filter replacing system, Ludl MAC5000. Fura-2 excitation and registration were recorded, using a 21HE filter set (Carl Zeiss, Berlin, Germany) with excitation filters BP340/30 and BP387/15, beam splitter FT-409 and emission filter BP510/90, objective lens Plan-Neo fluar 10×/0.3, excitation light source HBO 103W/2. Calcium responses were shown as a ratio of fluorescence intensities of Fura-2 excitation at 340 and 380 nm. To discriminate neurons and astrocytes, short-term application of 35 mM KCl at the end of experiments was used. This method was described in detail in our previous work [37,38]. Calibration for the maximum Ca^2+^-signal of neurons obtained from the cerebral cortex of control and Satb-knockout mice showed that the application of Ca^2+^ ionophore (Ionomycin) after inhibition of ATP synthesis (application FCCP and Oligomycin) induces Ca^2+^-signals differing in speed and amplitude in each individual neuron in experimental groups (Supplementary, Figure S2A–C). However, on average, the maximal [Ca^2+^]_i_ increase in control and at Satb-deletions differ by a few percent from each other (Supplementary, Figure S2D).

Therefore we determined the amplitudes of Ca^2+^ responses to activators of NMDAR, AMPAR, and KAR as (Δ)–Fmax-Fmin of Fura-2 fluorescence and an increase in Fura-2 fluorescence reflects a linear [Ca^2+^]_i_ increase in response to receptor agonists. ImageJ 2002 software (RRID: SCR_003070) was used to analyze data.

To analyze the development of the cell network, the cell culture was loaded with a Calcein AM fluorescent probe, which allows visualizing the cell bodies and their outgrowths. Calcein AM was loaded in HBSS solution with 10 HEPES, pH 7.4, at a final concentration of 5 μM at 37 °C for 40 min with subsequent 15 min washout. To register the fluorescence of Calcein, we used the system based on an inverted motorized microscope Leica DMI6000B with a high-speed monochrome CCD-camera HAMAMATSU C9100. For excitation and registration of Calcein fluorescence, we used an L5 filter cube (Leica Microsystems, Wetzlar, Germany) with BP 480/40 excitation filters, 505 dichromatic mirror, and BP 527/30 emission filter.

### 2.4. Extraction of RNA

MagMAX mirVana Total RNA Isolation Kit (Thermo Fisher Scientific, Cat. #A27828) was used for the extraction of total RNA. The RNA quality was estimated by electrophoresis in the presence of 1 μg/mL ethidium bromide (2% agarose gel in Tris/Borate/EDTA buffer). The concentration of the extracted RNA was determined with a NanoDrop 1000c spectrophotometer. RevertAid H Minus First Strand cDNA Synthesis Kit (Thermo Fisher Scientific, USA, Cat. #K1631) was used for reverse transcription of total RNA.

### 2.5. Real-Time Polymerase Chain Reaction (RT-qPCR)

Each PCR was performed in a 25 μL mixture composed of 5 μL of qPCRmix-HS SYBR (Evrogen, Moscow, Russia, Cat. #PK147L), 1 μL (0.2 μM) of the primer solution, 18 μL water (RNase-free), and 1 μL cDNA. DTlite Real-Time PCR System (DNA-technology, Moscow, Russia, 2017) was used for amplification. The amplification process consisted of the initial 5 min denaturation at 95 °C, 40 cycles of 30 s denaturation at 95 °C, 20 s annealing at 60–62 °C, and 20 s extension step at 72 °C. The final extension was performed for 10 min at 72 °C. The sequences of the primers used are presented in Table 1. All the sequences specific for the mouse were designed based on the analysis of the nucleotide sequences of the existing gene isoforms with FAST PCR 5.4 and NCBI Primer-BLAST software. The data were analyzed with DTlite software (DNA-technology, Moscow, Russia) and Origin 8.5 software (OriginLab Corporation, Northampton, MA, USA). The expression of the studied genes was normalized to gene encoding Glyceraldehyde 3-phosphate dehydrogenase (GAPDH) [39] and was presented relating control (neurons from Satb^fl/+^ * Nex^Cre/+^-mice). Data were analyzed using Livak’s method [40].

### 2.6. Statistical Analysis

All presented data were obtained from at least three cell cultures from 2–3 different passages. All values are given as mean ± standard error (SEM). Statistical analyses were performed by two-way ANOVA, followed by Sidak’s multiple comparison test or by paired t-test. MS Excel, ImageJ, Origin 2016 (OriginLab, Northampton, MA, USA), and Prism GraphPad 7 (GraphPad Software, RRID: SCR_002798) software were used for data and statistical analysis. In our experiments, we did not perform a predetermined sample size calculation to assess the effects. N—number of neurons that were analyzed in an experiment; n—number of the experiments.

## 3. Results

### 3.1. The Effects of Satb1 and Satb2 Deletions on the Expression of Neurotransmission-Regulating Genes

As shown by immunocytochemical staining of control cell cultures (Satb^+/+^ * Nex^Cre/+^) of the mouse cerebral cortex, the transcription factor Satb1 (Supplementary, Figure S3A) is expressed in 54.8 ± 16% of neurons identified using anti-NeuN antibodies. In this case, Satb1 is expressed in both GABA^+^ (Supplementary, Figure S3B) and GABA^-^-neurons. Satb2 is expressed in 42.3 ± 19% of neurons (Supplementary, Figure S3C), and its expression was not detected in GABA^+^-neurons (Supplementary, Figure S3D). Thus, the deletion of any of these transcription factors in a significant fraction of neurons in the mouse cerebral cortex will undoubtedly affect the functioning of neural networks.

**Figure 2:**
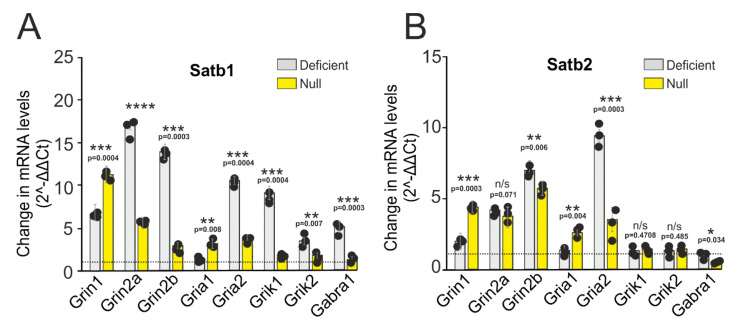
Deletion in Satb1 and Satb2 affects the expression of genes, encoding subunits of excitatory and inhibitory neurotransmitters produced by the cortical cell cultures. (**A**,**B**)—alteration in the expression of genes, encoding subunits of NMDAR (Grin1, Grin2a, Grin2b), AMPAR (Gria1, Gria2), KAR (Grik1, Grik2) and GABA(A)R (Gabra1) in the cortical neurons in Satb-deficient and Satb-null mice with deletions of transcription factors Satb1 (**A**) and Satb2 (**B**). Data obtained on four (*n* = 3) different cell cultures are presented. All values are given as mean ± SEM. Gene expression level was normalized to reference gene Gapdh and was presented relating control (neurons from Satb^+/+^ * Nex^Cre/+^-mice), which was considered as 1 (dashed line). Total RNA was obtained from 10 DIV cultures. Statistical analyses were performed by paired t-test. Comparison of gene expression was performed between Satb-deficient and Satb-null mice. The data were significant: n/s—data not significant (*p* > 0.05); * *p* < 0.05; ** *p* < 0.01; *** *p* < 0.001 and **** *p* < 0.0001.

Because deletions in both Satb1 and Satb2 cause disruption of brain functioning, we hypothesized that both proteins might be involved in the control of the synaptic transmission. In order to identify molecular effectors influencing neurotransmission downstream of Satb1 and Satb2, we used a candidate gene approach. Satb1 or Satb2 deficient neurons were cultured in vitro for ten days and were subjected to [Ca^2+^]_i_ dynamic analysis. Additionally, part of the cultures was used for mRNA isolation and subsequent RT-PCR analysis. We tested the expression levels of genes encoding various subunits of glutamate receptors. Using this approach, it is possible to determine more reliably the interaction between transcription factors and neurotransmission. The use of primary cell cultures for experiments on titration of receptor agonists and determination of EC50 values seem to be more optimal in comparison with cortical slices and organotypic cultures since by 10 days in vitro, the neuroglial network can be considered developed. However, the results obtained in vitro and in situ may differ in some details. In the cortical cell culture samples derived from deficient mice (Satb1^fl/+^ * Nex^Cre/+^) with Satb1 deletion, the expression of genes, *Grin2a, Grin2b, Grik1,* and *Grik2* encoding NMDAR and KAR subunits, increases by 17, 14.8, 7.6, and 4.6 times, respectively, compared to neurons from Satb^+/+^ * Nex^Cre/+^-mice (Figure 2A). An increase in the expression of the Grin2a and *Grin2b* genes is accompanied by an increase in the level of the *Grin1* gene encoding the GluN1-subunit of NMDAR by 6.3 and 12.3 times in Satb1-deficient and Satb1-null neurons, as compared to Satb1^+/+^ * Nex^Cre/+^, respectively (Figure 2A). Interestingly, the expression level of Gria2, encoding GluA2-subunit of AMPA receptor in Satb1^fl/+^ * Nex^Cre/+^-neurons increases by 10 times as compared to Satb1^+/+^ * Nex^Cre/+^, while the expression level of Gria1 (encoding GluA1-subunit) remains unchanged. In Satb1^fl/fl^ * Nex^Cre/+^-neurons the expression of Grin2a and Grin2b increases by 5.2 and 3.5 times, respectively, the expression of Gria1 and Gria2 increases by 3.7 and 3.9 times, respectively, and an alteration in the expression level of Grik1 and Grik2 is not reliable (Figure 2A) as compared to Satb1^+/+^ * Nex^Cre/+^-neurons.

Satb2 deletion also induces an increase in the expression level of *Grin2a* and *Grin2b* genes by 4 and 6.7 times, respectively, in the cortical neurons derived from Satb2^fl/+^ * Nex^Cre/+^-mice, and by 3.7 and 5.5 times in Satb2^fl/fl^ * Nex^Cre/+^ mice (Figure 2B). This change in gene expression is also accompanied by a 95% and 4.8-fold increase in the *Gria1* gene level in the Satb2^fl/+^ * Nex^Cre/+^ and Satb2^fl/fl^ * Nex^Cre/+^- mice, respectively (Figure 2B). Our results show that the expression level of *Gria1* gene remains unaltered as compared to the control group. Nevertheless, the expression level of the Gria 2 increases in Satb2^fl/+^ * Nex^Cre/+^- and Satb2^fl/fl^ * Nex^Cre/+^-neurons by 9.8 and 3.7 times, respectively (Figure 2B). Our findings demonstrate that deletion of Satb2 is associated with an unchanged expression level of *Grik1* and *Grik2* genes, encoding KAR as shown for Satb 1 deletion (Figure 2B).

As for Gabra 1, the gene encoding the inhibitory GABA receptor, its expression level increases in Satb1-deficient mice by 5.4 (Figure 2A) and decreases by 63% (Figure 2B) in Satb2-null mice, but in Satb1-null mice and Satb2-deficient.

**Figure 3:**
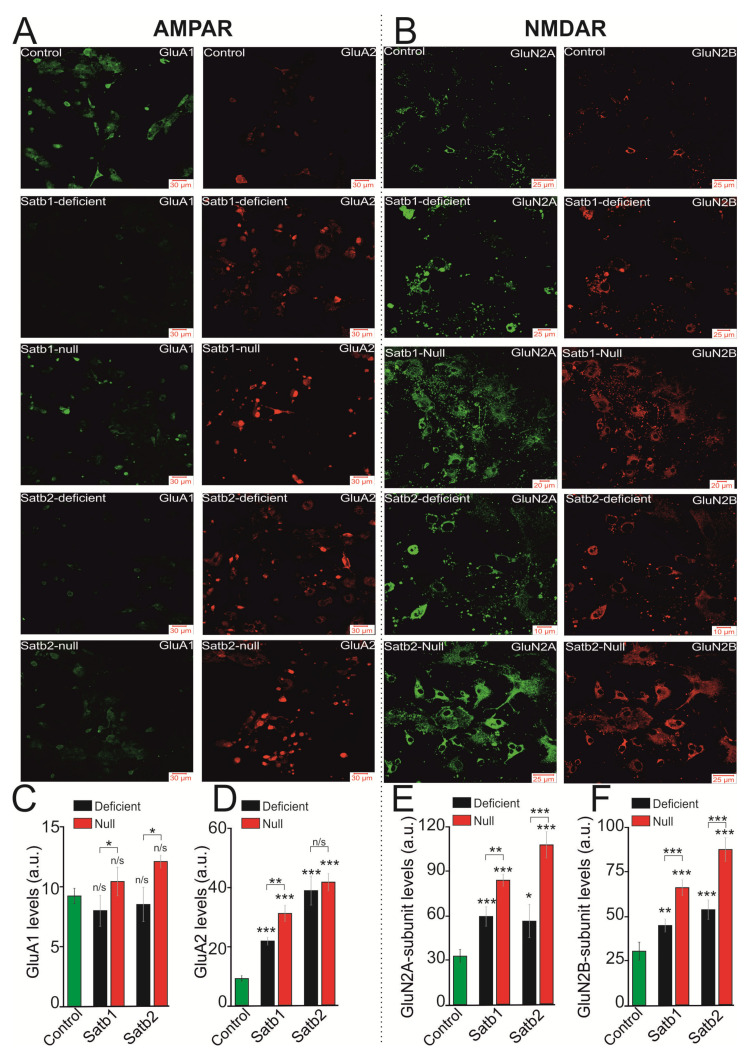
Expression of subunits forming AMPAR (GluA1, GluA2) and NMDAR (GluN2A, GluN2B) in neurons of the cerebral cortex derived from Satb-deficient, Satb-null, and control mice. (**A**,**B**)—Immunostaining of GluA1 (green color), GluA2 (red color) subunits of AMPA receptors (**A**) and GluN2A (green color), GluN2B (red color) of NMDA receptors (**B**) of cortical neurons from control (Satb^+/+^ * Nex^Cre/+^), Satb-deficient (Satb^fl/+^ * Nex^Cre/+^) and Satb-null (Satb2^fl/fl^ * Nex^Cre/+^) mice. (**C**–**F)**—The effects of Satb- and Satb2-deletions on the level of GluA1 (**C**), GluA2 (**D**), GluN2A (**E**), and GluN2B (**F**) subunits. Intensity levels of surface-expressed receptor subunits were determined by confocal imaging. We analyzed individual neurons which had fluorescence of Alexa Fluor 633 (GluA1, green color), Alexa Fluor 488 (GluA2, red color) and Alexa Fluor-555 (GluN2B, red color), and Alexa Fluor-594 (GluN2A, green color). The quantitative data reflecting the level of subunits expression are presented as fluorescence intensity values in summary bar charts (mean ± SEM). The values were averaged by 300 ± 50 neurons for each column. Statistical significance was assessed using paired t-test. The results obtained after immunostaining well agree with the data of fluorescent Ca^2+^ measurements presented in Figure 4 and Figure 5. After Ca^2+^ measurements, the cells were fixed and stained by the antibodies. We used the scans from three independent view fields for each experimental group. n/s—data not significant (*p* > 0.05); * *p* < 0.05; ** *p* < 0.01; and *** *p* < 0.001, comparing Satb-deficient group and Satb-null group with control mice. Significance indicated by a horizontal bar—comparison of Satb-deficient with Satb-null mice.

Staining of cell cultures with antibodies against the GluA1- and GluA2-subunits of AMPAR showed that the level of expression of the GluA1 protein in neurons from the cerebral cortex of Satb1-deficient and Satb1-null mice does not significantly change compared to control (Figure 3A,C). However, when comparing Satb1-deficient and Satb1-null neurons, it turned out that the level of the GluA1 subunit is 20% higher in Satb1-null (Figure 3A—green,C). As for the level of GluA2-subunits, with Satb1-deletion, there is a significant increase in their level by 67.8% and 71% in Satb1-deficient and Satb1-null neurons, compared to control, respectively (Figure 3A—red,D). Similarly, Satb2-deletion does not significantly alter the level of GluA1-subunits of the AMPAR in both Satb2-deficient and Satb2-null cases (Figure 3A,C). However, when comparing the Satb2-deficient group with the Satb2-null group, an increase in the level of the GluA1-subunit by 31.7% in the Satb2-null group was found (Figure 3C). As for the level of the GluA2-subunit of the AMPAR, in neurons from Satb2-deficient and Satb2-null mice, there is a significant increase of 81.6% and 78.4% (Figure 3A,D), respectively.

Immunocytochemical staining of cell cultures with antibodies against GluN2A- and GluN2B-subunits of the NMDAR showed that Satb1 deletion leads to an increase in the level of both subunits by 96% and 65.5% in the Satb1-deficient cortex and 1.8 times and 1.6 times in neurons from Satb1-null mice (Figure 3B,E,F) versus controls, respectively. Moreover, the level of GluN2A- and GluN2B-subunits is 44.3% and 55.4% higher in neurons from Satb1-null mice compared to Satb1-deficient, respectively (Figure 3E,F).

The amount of GluN2A- (Figure 3B—green) and GluN2B-subunits (Figure 3B—red) on the cell membrane of neurons with a Satb2 deletion is also higher than in control. In the cortical neurons from Satb2-deficient mice, the level of GluN2A- is higher by 83.8% (Figure 3E) and GluN2B- (Figure 3F) by 82.8%, and in Satb2-null by 3.2 times (Figure 3E) and 2.8 times (Figure 3F) compared to controls, respectively. In cortical neurons from Satb2-null mice, the levels of GluN2A- and GluN2B- are 73.7% and 59.8% higher, compared to neurons from Satb2-deficient mice (Figure 3E,F), respectively.

Therefore, deletions in Satb1 and Satb2 cause an increase in the expression level of genes encoding NMDAR subunits as compared to control. At the same time, deletions of these two transcription factors are associated with an increase in the expression level of genes encoding the AMPA receptor GluA2-subunit responsible for Ca^2+^-ion channel conductance. Changes in the expression level of genes encoding the NMDAR and AMPAR subunits in the Satb-knockout well correlate with the data of immunocytochemical staining of neurons, i.e., there is a reliable link between gene expression and encoding proteins. An increase in the expression level of genes encoding KAR subunits is seen only in the neurons of the cerebral cortex from Satb1-deficient mice, and in other cases, further research is needed to elucidate if the expression levels of Grik1 and Grik2 genes change or remain constant (Figure 2A,B). As to inhibitory neurotransmission, it has been shown that the expression level of the *Gabra1* gene in the neurons derived from mice with Satb1 knockout is higher than in those with Satb2 deletion.

### 3.2. Deletions in Satb1 and Satb2 Cause an Increase in the Sensitivity of NMDA Receptor to the Agonist and Contribute to Higher Amplitude of Ca^2+^-Signals Generated by Cortical Neurons

**Figure 4:**
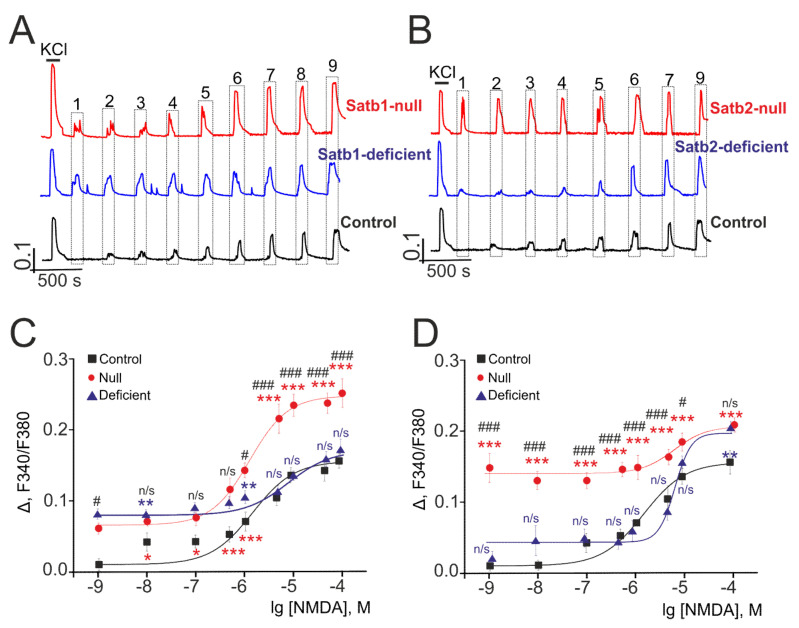
The effect of deletion in the transcription factors Satb1 and Satb2 on the Ca^2+^-signals produced by cortical neurons after activation of NMDA-receptors. (**A**,**B)** application of NMDA in Mg^2+^-free medium to the neurons of brain cortex isolated from the Satb1- (**A**) and Satb2- knockout mice (**B**). (**C**,**D**) dependence of the Ca^2+^-responses amplitude on NMDA concentration in the brain cortex neurons of the Satb1 (**C**) and Satb2 (**D**) knockout mice. NMDA concentrations are specified with horizontal lines accompanied with correspondent cyphers: 1–0.001, 2–0.01, 3–0.1, 4–0.5, 5–1, 6–5, 7–10, 8–50, 9–100 µM. For each concentration, average data are represented, obtained from several hundreds of neurons in 5 independent experiments ± SEM. For panels (**A**,**B**), averaged Ca^2+^-responses for neurons from individual experiments are represented. For panels (**A**,**B**), we analyzed 200 cells (*n* = 200) in each experiment. For panels (**C**,**D**), every experiment was repeated six times (*n* = 6) using cell cultures from separate passages. Statistical analyses were performed by two-way ANOVA, followed by Sidak’s multiple comparison test. * *p* < 0.05, ** *p* < 0.01, and *** *p* < 0.001 comparing Satb-deficient group (blue asterisk) and Satb-null group (red asterisk) with control mice; # *p* < 0.05 and ### *p* < 0.001 comparing Satb-deficient group with Satb-null group; n/s (blue) and n/s (black)—data not significant in experimental Satb-deficient group compared to control mice, and Satb-deficient group compared to Satb-null group, respectively.

Our earlier study reported that deletion in the transcription factor Sip1 in the cerebral cortex is associated with alterations in the activity of NMDAR and AMPAR, except for KAR [41]. To elucidate the role of deletion in transcription factors Satb1 and Satb2 in the regulation of the activity of ionotropic glutamate receptors responsible for excitation of neural cells, Ca^2+^-sensing probe (Fura-2) was used to measure intracellular [Ca^2+^]_i_ in the neurons of the cortical cultures in response to short-term (30 s) activation of glutamate receptors by selective agonists—NMDA, 5-Fluorowillardiine (FW), and Domoic acid (DA). Figure 4 represents averaged Ca^2+^-signals of several hundred cells of the neurons derived from the cerebral cortex of control (Satb1^+/+^ * Nex^Cre/+^), Satb1^fl/+^ * Nex^Cre/+^, and Satb1^fl/fl^ * Nex^Cre/+^ mice with Satb1 knockout in response to different NMDA concentrations in Mg^2+^-free medium. The basal level of [Ca^2+^]_i_ in the neurons of the cerebral cortex obtained from different mice is 0.24 ± 0.04 a.u. and is not different in mice with Satb1-, Satb2- knockouts and control mice. Ca^2+^-signals are produced by the cortical neurons in Satb1^fl/fl^ * Nex^Cre/+^- and Satb1^fl/+^ * Nex^Cre/+^-mice in response to 0.001 µM NMDA (Figure 4A, red and blue curves), while neurons derived from the control mice produce no signal in response to the above concentration of NMDAR agonist (Figure 4A, black curve) but begin to generate Ca^2+^-signals with small signal amplitude in response to 0.01 µM NMDA.

Analysis of the amplitude of Ca^2+^-signals produced by neurons derived from mice with Satb1 knockout in response to NMDA treatment showed that half of the maximal (EC5) increase of [Ca^2+^]_i_ is 1.26 ± 0.04 µM (Hill coefficient (n) 1 ± 0.05) and 9.94 ± 0.06 µM (*n* = 0.97 ± 0.03) in neocortical neurons from Satb1^fl/fl^ * Nex^Cre/+^ and Satb1^fl/+^ * Nex^Cre/+^ mice, respectively, and the EC50 value for the neurons from Satb1^+/+^ * Nex^Cre/+^-mice is—6.39 ± 0.09 µM (*n* = 0.98 ± 0.05) (Figure 4C). On the whole, the amplitude of Ca^2+^-signals is, on average, higher in the neurons of the cerebral cortex from mice with Satb1 knockout and only when cells are treated with NMDA in the concentration range from 5 to100 µM, Ca^2+^-signals produced by neurons from control mice are comparable to the amplitude of the Ca^2+^-signals produced by neurons from Satb1^fl/+^ * Nex^Cre/+^ mice. The amplitude of Ca^2+^- signals generated by neurons from Satb1^fl/fl^ * Nex^Cre/+^ and Satb1^fl/+^ * Nex^Cre/+^ mice in response to maximal NMDA concentration (100 µM) is 0.254 and 0.153, respectively, on average, it is higher than control—0.139 (Table 2).

Deletion in the transcription factor Satb2 has a similar effect on Ca^2+^-signals produced by the cortical neurons after NMDA receptor activation. The high amplitude of Ca^2+^-signals in response to NMDA concentration of 0.001 µM is typical for neurons derived from the cortex of Satb2^fl/fl^ * Nex^Cre/+^-mice with Satb2 knockout (Figure 4B, red curve), and an increase in agonist concentration results in negligible increment to the amplitude of Ca^2+^-signals. Neurons, derived from Satb2^fl/+^ * Nex^Cre/+^ -mice similar to neurons from Satb2^fl/fl^ * Nex^Cre/+^-mice produce Ca^2+^-signals with higher amplitude in response to treatment with 0.001 µM (Figure 4B, blue curve, symbol 1) but such an increase in the amplitude of [Ca^2+^]_i_ is lower by several times. The EC50 value for Satb2^fl/fl^ * Nex^Cre/+^-neurons is 2.83 ± 0.04 µM (*n* = 0.97±0.06); the EC50 value for Satb2-deficient-neurons is 6.19 ± 0.08 µM (*n* = 1.04 ± 0.02), which is close to 6.39 ± 0.09 µM (*n* = 0.98 ± 0.05), the value for neurons from control mice (Figure 4D), on the whole, sigmoidal curves, that define the dependence of the amplitude of Ca^2+^-signals produced by the neurons from mice with Satb2- knockout on the NMDA concentration, are higher than the amplitude of Ca^2+^-signals from control mice. The average amplitudes of Ca^2+^-signals of Satb1^fl/fl^ * Nex^Cre/+^ and Satb1^fl/+^ * Nex^Cre/+^ -neurons in response to the maximum concentration of NMDA are 0.173 and 0.184, respectively (Table 2). These values are greater than those for control neurons. At the same time, Ca^2+^-signals Satb1^fl/fl^ * Nex^Cre/+^ and Satb2^fl/fl^ * Nex^Cre/+^ -neurons for both low (0.001 µM) and high (10 µM) NMDA concentrations were completely suppressed by the selective antagonist, D-AP5 (Supplementary, Figure S11).

Therefore, deletions in the transcription factors Satb1 and Satb2 in the cerebral cortex is associated with increased sensitivity of the cortical neurons from null and deficient mice to NMDAR activation resulting in the lower EC50 values and higher amplitudes of Ca^2+^-signals in response to NMDA treatment in Mg^2+^-free medium as compared to that of cortical neurons from control mice.

### 3.3. Deletions in Satb1 and Satb2 Reduce the Sensitivity of the AMPA Receptors of the Cortical Neurons to the Agonist

**Figure 5:**
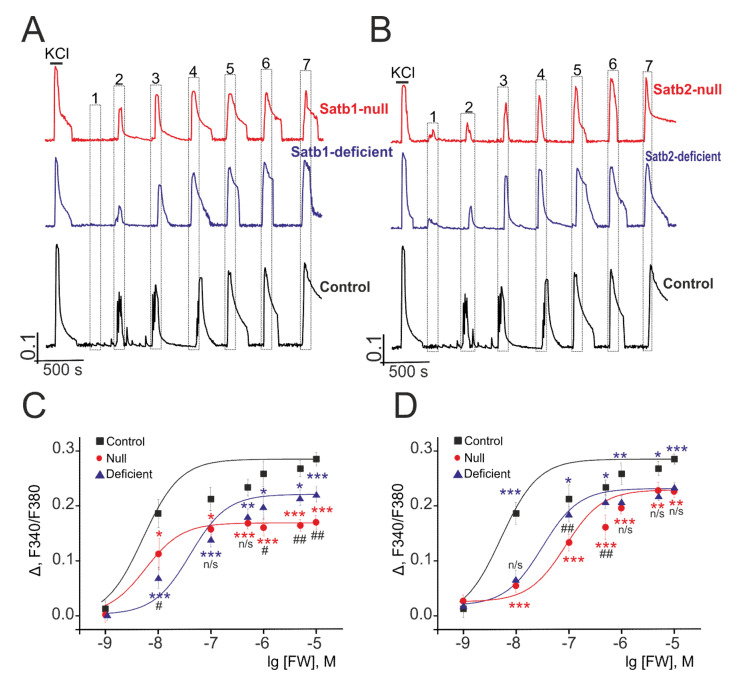
The effect of deletion in the transcription factors Satb1 and Satb2 on the Ca^2+^-signals produced by cortical neurons after activation of AMPA receptors. (**A**,**B**)—application of enhancing FW concentrations in the presence of an inhibitor for desensitization of the AMPA receptors, cyclothiazide (CTZ, 5 µM), to the neurons of brain cortex isolated from the Satb1 (**A**) and Satb2 (**B**) knockout mice. (**C**,**D**)—dependence of the Ca^2+^-responses amplitude on FW concentration in the cortical neurons from Satb1 (**C**) and Satb2 (**D**) knockout mice. FW concentrations are specified with horizontal lines accompanied with correspondent cyphers: 1–0.001, 2–0.01, 3–0.1, 4–0.5, 5–1, 6–5, 7–10 µM. For each concentration, average data are represented, obtained from several hundreds of neurons in 5 independent experiments ± SEM. For panels (**A**,**B**), averaged Ca^2+^-responses for neurons from individual experiments are represented. For panels (**A**,**B**), we analyzed 200 cells (*n* = 200) in each experiment. For panels (**C**,**D**), every experiment was repeated five times (*n* = 5) using cell cultures from separate passages. Statistical analyses were performed by two-way ANOVA, followed by Sidak’s multiple comparison test. * *p* < 0.05, ** *p* < 0.01, and *** *p* < 0.001 comparing Satb-deficient group (blue asterisk) and Satb-null group (red asterisk) with control group; # *p* < 0.05 and ## *p* < 0.01 comparing Satb-deficient group with Satb-null group; n/s (blue) and n/s (black)—data not significant in experimental Satb-deficient group compared to control group, and Satb-deficient group compared to Satb-null group, respectively.

As AMPA-receptors are highly dynamic proteins [42], these proteins have been activated with 5-Fluorowillardiine (FW, Alomone Labs, Jerusalem, Israel, Cat. #F-205), a selective agonist, in the presence of cyclothiazide (CTZ, Tocris Cookson, Bristol, UK, Cat. #0713), a desensitization inhibitor, applied before FW, in order to get maximal Ca^2+^-signals. Neurons in the cerebral cortex derived from control mice generate increased [Ca^2+^]_i_ in response to 0.01 µM FW and the amplitude of the signal is higher than that from the neurons derived from Satb1^fl/+^ * Nex^Cre/+^ and Satb1^fl/fl^ * Nex^Cre/+^ mice (Figure 5A). In response to high FW concentrations, the amplitudes of Ca^2+^-signals of cortical neurons from mice with knockout are also lower than those from control mice (Table 2). For activation of the AMPA receptors in the neurons from Satb1^+/+^* Nex^Cre/+^-mice, Satb1^fl/+^* Nex^Cre/+^ and Satb1^fl/fl^ * Nex^Cre/+^-mice the EC50 value should be 0.004 ± 0.0003 µM (*n* = 0.98 ± 0.04) (Figure 5C, black curve), 0.018 ± 0.001 µM (*n* = 1.14 ± 0.06), and 0.002 ± 0.0004 µM (*n* = 1.12 ± 0.03), respectively (Figure 5C, blue and red curves).

Low sensitivity of the AMPA receptors to activation of the selective agonist is typical for the neurons in the cerebral cortex from mice with Satb2 knockout, the amplitudes of Ca^2+^-signals in response to the addition of various FW concentrations are lower than those generated by neurons from Satb2^+/+^* Nex^Cre/+^-mice (Figure 5B, Table 2). The EC50 value is 0.025 ± 0.003 µM (*n* = 1.2 ± 0.07) and 0.092 ± 0.006 µM (*n* = 1.1 ± 0.02) for neurons from mice with Satb2^fl/+^ * Nex^Cre/+^ and Satb2^fl/fl^ * Nex^Cre/+^ knockout; it is greater by a factor of 5 and 23, respectively than that for neurons from Satb2^+/+^ * Nex^Cre/+^-mice (Figure 5D).

Thus, when comparing the activity of AMPAR neurons in the cortical neurons from mice with a knockout in Satb1 and Satb2 transcription factors and from control mice, it is shown that these deletions affect AMPAR activity, decreasing the sensitivity of these receptors to selective agonists, resulting in the lower amplitudes of Ca^2+^-signals in response to the agonist the EC50 value for which becomes greater. Interestingly, Satb-deletion leads to an increase in the level of expression of the *Gria2* gene and the encoded GluA2-subunit AMPAR, in the presence of which the receptor channel is practically impermeable to Ca^2+^ ions. Repeated applications of the maximal (10 μM) FW concentration to the cortical neurons lead, on average, to an almost complete repetition of the amplitude and shape of Ca^2+^-responses both in control and in cells from Satb1-null and Satb2-null mice. Incubation of cortical neurons obtained from control mice with 100 μM 1-Naphthyl acetyl spermine (NASPM, 100 μM), an antagonist of the calcium-conductive AMPA-receptors, leads to a decrease in the amplitude of the Ca^2+^-signal upon application of 10 μM FW to the level of neuronal Ca^2+^-responses derived from Satb-null mice (Supplementary, Figure S4). This data suggest the important role of increased expression of the GluA2-subunit of AMPAR in the limiting increase of [Ca^2+^]_i_ upon complete deletion of both Satb transcription factors. At the same time, the effect of NASPM on the cortical neurons from Satb1-null and Satb2-null mice is much less pronounced and, probably, is associated with a small number of Ca^2+^-conducting GluA2-lacking AMPARs on their membranes.

### 3.4. Deletions in Satb1 and Satb2 have Different Effects on the Sensitivity of KA Receptors to a Selective Agonist

**Figure 6:**
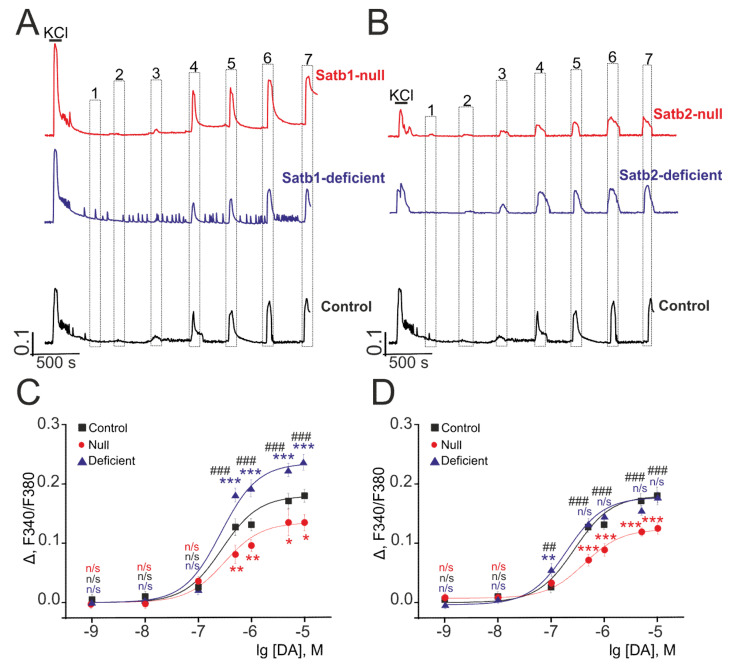
The effect of deletion in the transcription factors Satb1 and Satb2 on the Ca^2+^-signals produced by cortical neurons after activation of kainate receptors. (**A**,**B**) application of enhancing concentrations of KA-receptors activator, domoic acid (DA) in the presence of a selective antagonist of the AMPA-receptors, GYKI-52466 (30 µM) and an inhibitor for desensitization of the KA-receptors, concanavalin A (ConA, 200 µg/mL) to the neurons of brain cortex isolated from the Satb1 (**A**) and Satb2 (**B**) knockout mice. (**C**,**D**) dependence of the Ca^2+^-responses amplitude on DA concentration in the cultured cortical neurons from Satb1 (**C**) and Satb2 (**D**) knockout mice. DA concentrations are specified with horizontal lines accompanied with correspondent cyphers: 1–0.001, 2–0.01, 3–0.1, 4–0.5, 5–1, 6–5, 7–10 µM. For each concentration, average data are represented, obtained from several hundreds of neurons in 5 independent experiments ± SEM. For panels (**A**,**B**), averaged Ca^2+^-responses for neurons from individual experiments are represented. For panels (**A**,**B**), we analyzed 200 cells (*n* = 200) in each experiment. For panels (**C**,**D**), every experiment was repeated four times (*n* = 4) using cell cultures from separate passages. Statistical analyses were performed by two-way ANOVA, followed by Sidak’s multiple comparison test. * *p* < 0.05, ** *p* < 0.01, and *** *p* < 0.001 comparing Satb-deficient group (blue asterisk) and Satb-null group (red asterisk) with control group; ## *p* < 0.01, and ### *p* < 0.001 comparing Satb-deficient group with Satb-null group; n/s (blue) and n/s (black)—data not significant in experimental Satb-deficient group compared to control group, and Satb-deficient group compared to Satb-null group, respectively.

Neuronal hyperactivity, which could be induced by glutamate and KA, was a hallmark of epileptic disorders [43]. Addition of Domoic acid (DA, Tocris, Cat. #0269), a selective agonist of KA, in the presence of GYKI-52466 (Tocris, Cat. #1454), AMPAR antagonist (30 µM), and concanavalin A (Sigma-Aldrich, Cat. #C5275), an inhibitor for desensitization of the KA-receptors, stimulates Ca^2+^-signals from neurons in the cultured cortical neurons from control mice in response to the concentration of an activator (0.1 µM) (Figure 6A, black curve), as well as from neurons, derived from null and deficient mice with knockouts in Satb1 (Figure 6A, red and purple curves) and Satb2 (Figure 6B, red and purple curves). An increased DA concentration promotes an almost linear increase of the amplitudes of Ca^2+^-signals generated by the neurons from control and knockout mice. The amplitudes of Ca^2+^-signals produced by neurons from control mice, null mice, and deficient Satb1-knockout mice in response to maximum (10 µM) DA concentration are 0.181, 0.239, and 0.137, respectively. Herewith, the EC50 values are 0.32 ± 0.003 µM (*n* = 0.89 ± 0.12) for neurons from control mice, 0.27 ± 0.003 µM (*n* = 0.95 ± 0.06) for Satb1^fl/fl^ * Nex^Cre/+^, and 0.39± 0.002 µM (*n* = 1.04 ± 0.03) for Satb1^fl/+^ * Nex^Cre/+^ (Figure 6C, Table 2). Cortical neurons from Satb2-targeted mice, on average, generate Ca^2+^-signals with the amplitude which is lower than that typical to neurons from Satb2^+/+^* Nex^Cre/+^ -mice even in response to maximum DA concentration, 0.126 is the amplitude for Satb2^fl/fl^ * Nex^Cre/+^ and 0.179—for Satb2^fl/+^* Nex^Cre/+^. Nevertheless, the EC50 value for activation of KAR neurons from Satb2^fl/+^* Nex^Cre/+^-mice is less than that for Satb2^+/+^* Nex^Cre/+^-mice and accounts for 0.176 ± 0.004 µM (*n* = 1.2 ± 0.05) taking into account that the amplitudes of Ca^2+^ signals are virtually the same (Figure 6C, Table 2). For KAR neurons from Satb2^fl/fl^ * Nex^Cre/+^-mice, the EC50 values is 0.49 ± 0.005 µM (*n* = 1.2 ± 0.03) (Figure 6C, Table 2).

Taken together, deletion in Satb1 transcription factor leads to higher amplitudes of Ca^2+^-signals in response to KAR activation in cortical neurons from Satb1^fl/+^ * Nex^Cre/+^-mice. Simultaneously, we observe a decline in Ca^2+^-signals of the neurons from Satb2^fl/fl^ * Nex^Cre/+^-mice when compared to those in Satb1^fl/+^ * Nex^Cre/+^ and control mice. For instance, Satb2 deletion causes a decrease in the amplitudes of Ca^2+^signals in response to DA in cortical neurons from Satb2^fl/fl^ * Nex^Cre/+^ and Satb2^fl/+^ * Nex^Cre/+^ mice when compared to control mice and Satb1 knockout mice. These results suggest that deletions in these homologous transcription factors have a somewhat multifaceted effect.

The above-shown patterns of neuronal Ca^2+^-signals in response to excitatory glutamate receptor agonists persist against the background of selective receptor antagonists (Supplementary, Figure S5). Application of increasing concentrations of NMDA in a magnesium-free medium with the addition of an AMPA-receptor antagonist, GYKI-52466 (30 µM), still produces high Ca^2+^-signals in Satb1- (Supplementary, Figure S5A) and Satb2-null (Supplementary, Figure S5B) neurons. Similarly shown for AMPA-receptors, when FW application was performed with NMDAR antagonist, D-AP5 (10 μM), neurons with the Satb1- and Satb2-deletions showed smaller amplitudes of [Ca^2+^]_i_-increases (Supplementary, Figure S5C,D), which is consistent with the data presented in Figure 5. AMPAR (GYKI-52466, 30 µM) and NMDAR (D-AP5 10 µM) (Supplementary, Figure S5E,F) were inhibited when KAR was activated by a selective agonist, which also confirmed the Ca^2+^-signaling patterns shown in Figure 6 for neurons with deletion of transcription factors Satb1 and Satb2.

### 3.5. Deletions in Satb1 and Satb2 Suppress the Development of the Inhibitory System of Neurotransmission in the Cortical Neurons In Vitro

As shown above, Satb1 and Satb2 deletions affect the activity of the glutamatergic signaling system, and in further experiments, we attempted to investigate the development of the GABAergic system during the development of the neuronal network.

**Figure 7:**
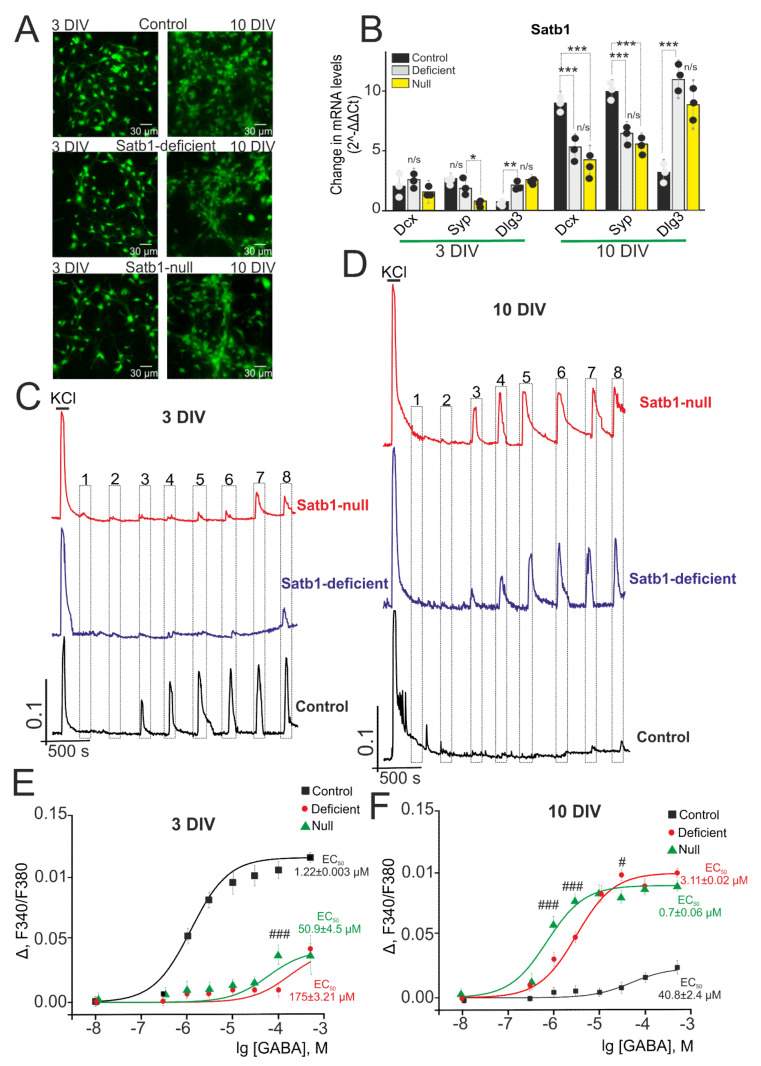
The effect of deletion in the transcription factor Satb1 on Ca^2+^-signals from cortical immature and mature neurons in response to activation of GABA receptors. (**A**)—morphology of a cortical cell culture obtained from mice with a deletion of the transcription factor Satb1 during cultivation in vitro. The cells are loaded with the Calcein AM probe. (**B**) changes in the expression of genes encoding developmental markers during culturing of cortical cells obtained from mice with a Satb1 deletion in comparison with the control group (Satb1^+/+^ * Nex^Cre/+^). Differences in the expression of all genes between 3 DIV and 10 DIV are significant at *p* < 0.001 (***). Significance between groups means n/s—*p* > 0.05, *—*p* < 0.05, ** *p* < 0.01, and *** *p* < 0.001. (**C**,**D**)—application of enhancing concentrations of GABA-receptors activator, γ-aminobutyric acid (GABA) to the neurons of brain cortex isolated from the Satb1 knockout mice on the 3 (**C**) and 10 (**D**) day in vitro. (**E**,**F**) dependence of the Ca^2+^-responses amplitude on GABA concentration in the cultured cortical neurons from Satb1 knockout mice in the third (**E**) and tenth (**F**) day of cultivation. GABA concentrations are specified with horizontal lines accompanied with correspondent cyphers: 1–0.01, 2–0.3, 3–1, 4–3, 5–10, 6–30, 7–100, 8–500 µM. For each concentration, average data are represented, obtained from several hundreds of neurons in 5 independent experiments ± SEM. For panels (**C**,**D**), averaged Ca^2+^-responses for neurons from individual experiments are represented. For panels (**C**,**D**), we analyzed 200 cells (*n* = 200) in each experiment. For panels (**E**,**F**), every experiment was repeated five times (*n* = 5) using cell cultures from separate passages. Statistical analyses were performed by two-way ANOVA, followed by Sidak’s multiple comparison test. Differences between groups Satb1-deficient, Satb1-null compared with control (Satb1^+/+^* Nex^Cre/+^) for panels (**E**,**F**) are significant *** *p* < 0.001 (not indicated), # *p* < 0.01, and ### *p* < 0.001 comparing Satb1-deficient group with Satb1-null group; in other cases, comparisons of Satb1-deficient vs. Satb1-null—are not significant (not indicated).

During the cultivation of cells isolated from Satb1-deficient and Satb1-null mice, the development of the cellular network occurred, which was expressed in an increase in the density of neurites from 3 DIV to 10 DIV (Figure 7A). At the same time, visually, the density of the network was slightly lower in the culture of the cerebral cortex isolated from Satb1-deficient and Satb1-null mice, as compared to the control (Satb1^+/+^* Nex^Cre/+^). This difference could be related to the influence of the Satb1 deletion on the development of neuroplasticity. It was shown that the development of the cerebral cortex in Satb1-null mice proceeded without significant disturbances. However, for pyramidal neurons in the postnatal period, a decrease in the density of dendritic spines, playing a key role in synaptic transmission and plasticity, was shown, as indicated by Balamotis et al. [44]. At the same time, the analysis of the expression of genes encoding neuronal developmental markers showed that during cell cultivation, there was a significant increase in the expression of *Dcx*, *Syp*, and *Dlg3* genes encoding Doublecortin, Synaptophysin, and PSD-95, respectively (Figure 7B). Thus, by 10 DIV, the expression level of Dcx, Syp, and Dlg3 increased in the Satb1-deficient group by 2.2, 3.2, and 5.4 times, respectively, compared with the expression level at 3 DIV. In the Satb1-null group, the expression of Dcx, Syp, and Dlg3 also increased 3.1, 3.6, and 3.3 times compared with 3 DIV (Figure 7B). In control cells (Satb1^+/+^ * Nex^Cre/+^) by 10 DIV, the expression level of the studied genes increased by 4.6, 4.5, and 3.1 times for Dcx, Syp, and Dlg3, respectively (Figure 7B). It can be seen that the level of expression of genes encoding Doublecortin and Synaptophysin is higher on the 10 DIV in control compared to cortical cultures from Satb1-deficient and Satb1-null mice (Figure 7B). Thus, cells from Satb1-deficient and Satb1-null mice lag behind the control in terms of developmental gene expression, except for *Dlg3* expression. At the same time, the expression of the inhibitory synaptic marker protein, vesicular GABA transporter (VGAT), is at a low level both in control and in cortical cultures from Satb1-deficient and Satb1-null mice by 3 DIV (Supplementary, Figure S6A) with some trend towards a decrease in Satb1-knockout. However, already by 10 DIV, there is a significant increase in the amount of VGAT in control neurons and Satb1-deficient neurons (Supplementary, Figure S6B), and in cells from Satb1-null mice, a noticeably lower expression of VGAT is observed (Supplementary, Figure S6B—bottom line). Consequently, in the process of culturing neurons of the cerebral cortex from Satb1-knockout mice, an inhibitory component of the network also develops an increase in VGAT expression, but also with some lag in this process in Satb1-null neurons.

Figure 7 shows the amplitudes of Ca^2+^-signals from immature (3 DIV, C) and mature (10 DIV, D) neurons derived from the cerebral cortex of Satb1^+/+^* Nex^Cre/+^-mice (control) and Satb1-knockout mice. Immature Satb1^+/+^* Nex^Cre/+^-neurons produce signals with high amplitudes (0.053) in response to GABA application (1 µM) (Figure 7C, black curve), while neurons from the cerebral cortex from Satb1-deficient- and Satb1-null-mice generate Ca^2+^-signals with the amplitude of 0.008 and 0.01, respectively (Figure 7C, purple and red curves), that is by 30–45% higher than the [Ca^2+^]_i_ fluctuation level at rest. In contrast to Ca^2+^-signals of Satb1^+/+^ * Nex^Cre/+^-neurons with the amplitude of 0.1 ± 0.02, an increase in GABA concentration up to 100 µM and 500 µM causes distinct Ca^2+^-signals with the amplitudes (0.044) and (0.045) for Satb1-null- and Satb1-deficient-neurons, respectively. Analysis of the dependencies of the amplitudes of Ca^2+^-signals of the cortical neurons on GABA concentration shows that the EC50 value for Satb1^+/+^ * Nex^Cre/+^-neurons after 3 day cultivation is 1.22 ± 0.003 µM (*n* = 1.1 ± 0.01), 175 ± 3.21 µM (*n* = 0.89 ± 0.14) for Satb1-deficient-neurons and 50.9 ± 4.5 µM (*n* = 0.94 ± 0.09) for Satb1-null-neurons (Figure 7E).

Cultivation of cortical neurons derived from Satb1^+/+^ * Nex^Cre/+^-mice up to 10 DIV leads to the fact that application of only high GABA concentrations (100–300 µM) results in Ca^2+^-signals with low-amplitude (0.017–0.024) (Figure 7D, black curve). However, at the same time, there is an increase in the amplitude of Ca^2+^-signals in neurons with Satb1 knockout—Satb1-deficient and Satb1-null neurons produce signals with high amplitudes (0.03 and 0.06), respectively (Figure 7D, purple and red curves) after GABA application (1 µM). A successive increase of GABA concentration leads to a rise in the amplitude of increased [Ca^2+^]_i_ in neurons with deletion. For Satb1^+/+^* Nex^Cre/+^-neurons the EC50 value increases to activate GABA receptors during the development and aging of the neural network (EC50 = 40.8 ± 2.4 µM; *n* = 1.1 ± 0.07). However, for Satb1-deficient- and Satb1-null neurons, this value decreases and accounts for 3.11 ± 0.02 µM (*n* = 0.91 ± 0.1) and 0.7 ± 0.06 µM (*n* = 1.01 ± 0.04), respectively (Figure 7F).

**Figure 8:**
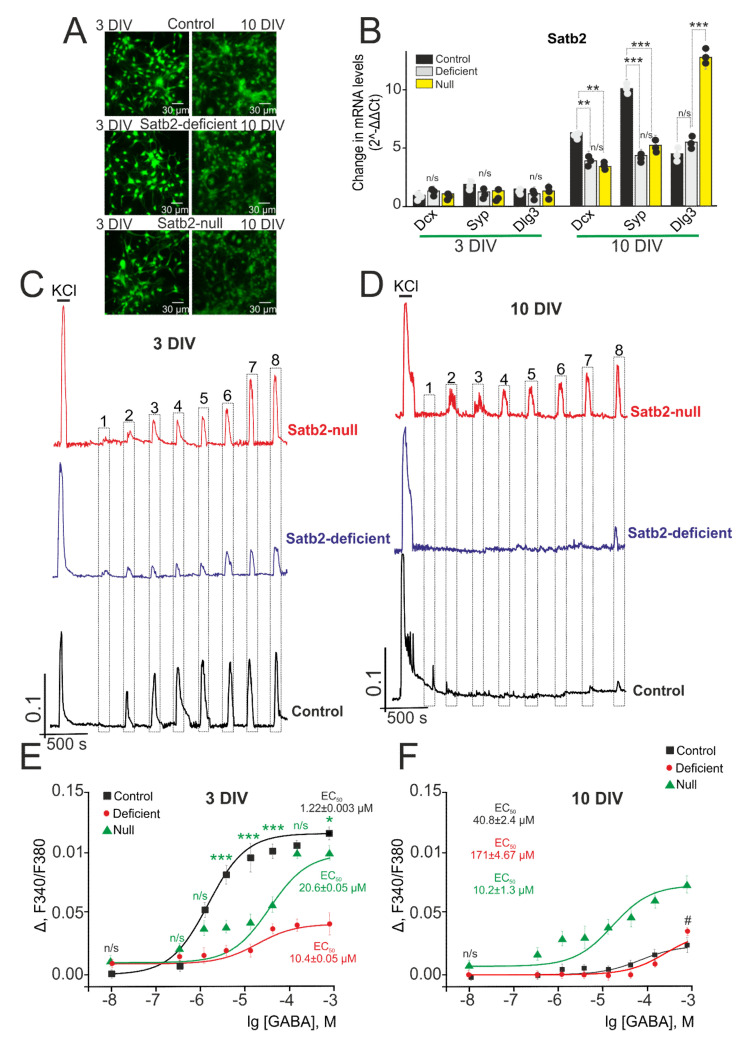
The effect of deletion in the transcription factor Satb2 on Ca^2+^-signals from cortical immature and mature neurons in response to activation of GABA receptors. (**A**) Morphology of a cortical cell culture obtained from mice with a deletion of the transcription factor Satb2 during cultivation in vitro. The cells are loaded with the Calcein AM probe. (**B**) Changes in the expression of genes encoding developmental markers during culturing of cortical cells obtained from mice with a Satb2 deletion in comparison with the control group (Satb2^fl/+^ * Nex^Cre/+^). Differences in the expression of all genes between 3 DIV and 10 DIV are significant at *p* < 0.001 (***). Significance between groups means n/s—*p* > 0.05, * *p* < 0.05, ** *p* < 0.01, and *** *p* < 0.001. (**C**,**D**) application of enhancing concentrations of GABA receptors activator, γ-aminobutyric acid (GABA) to the neurons of brain cortex isolated from the Satb2 knockout mice on the 3 (**C**) and 10 (**D**) day in vitro. (**E**,**F**) dependence of the Ca^2+^-responses amplitude on GABA concentration in the cultured cortical neurons from Satb2 knockout mice in the third (**E**) and ten (**F**) day of cultivation. GABA concentrations are specified with horizontal lines accompanied with correspondent cyphers: 1–0.01, 2–0.3, 3–1, 4–3, 5–10, 6–30, 7–100, 8–500 µM. For panels (**A**,**B**), we analyzed 200 cells (*n* = 200) in each experiment. For panels (**E**,**F**), every experiment was repeated five times (*n* = 5) using cell cultures from separate passages. Statistical analyses were performed by two-way ANOVA, followed by Sidak’s multiple comparison test. For panel (**E**), the differences between Satb2-deficient vs. control and Satb2-deficient vs. Satb2-null group are significant *** *p* < 0.001 (not indicated); Satb2-deficient group vs. control group values are significant * *p* < 0.05, *** *p* < 0.001. For panel F, the differences between Satb2-null group vs. control group and Satb2-null group vs. Satb2-deficient group are significant *** *p* < 0.001 (not indicated); Satb2-deficient group vs. control values are significant * *p* < 0.05, in other cases the comparisons of Satb2-deficient vs. control group are not significant (not indicated). n/s—differences are not significant.

Likewise, the Satb2 deletion did not significantly alter the morphology of the cortical cell culture. As shown in Figure 8A, the number of cells on 3 DIV did not significantly differ between the studied groups, and by 10 DIV there was a comparable increase in the density of cell processes both in the Satb2-deficient and Satb2-null groups and in the control (Satb2^+/+^ * Nex^Cre/+^). By 10 DIV in the control group (Satb2^+/+^ * Nex^Cre/+^), the expression level of genes encoding Dcx, Syp, and Dlg3 increased by 4.7, 4.1, and 2.7 times, respectively (Figure 8B). At the same time, in the experimental groups Satb2-deficient and Satb2-null, there was a less pronounced increase in the expression of genes *Dcx* and *Syp*, compared with the control, by 2 and 2.6 times in the Satb2-deficient group and 3.1 and 2 times in the Satb2-null group (Figure 8B). Whereas the level of Dlg3 in the Satb2-deficient and Satb2-null group cells is 3.1 and 7.3 times higher compared to 3 DIV and 10 DIV in control. These data on the analysis of images of cell cultures indicated a similar level of development of neuroglial cell network of the cerebral cortex with a Satb2 deletion during cultivation. However, there is a 10 DIV lag in the expression of developmental markers as compared to control cells. As for the level of VGAT in cortical neurons from Satb2-knockout mice, immunocytochemical staining did not reveal significant differences between Satb2-deficient, Satb2-null, and control by 3 DIV, when VGAT immunoreactivity was detected only in the processes of single neurons (Supplementary Figure S7A). Cell cultivation up to 10 DIV leads to the development of a network and the formation of connections between neurons, which is accompanied by an increase in the amount of VGAT both in control and in cells from Satb2-knockout mice (Supplementary, Figure S7B), without significant differences.

In immature (3 DIV) neurons from mice with Satb2-deficient and Satb2-null neurons the amplitudes of Ca^2+^-signals are 0.09 and 0.01 even after GABA application (0.01 µM) (Figure 8C, purple and red curves), while neurons from control (Satb2^+/+^ * Nex^Cre/+^)-mice produce increased [Ca^2+^]_i_ only after GABA application (1 µM) (Figure 8C, black curve). For activation of GABA receptors in immature neurons from mice with Satb2-deficient and Satb2-null mice, the EC50 value is 10.4 ± 0.05 µM (*n* = 1.12 ± 0.07) and 20.6 ± 0.06 µM (*n* = 0.89 ± 0.14), respectively, it is greater than (1.22 ± 0.003 µM; *n* = 1.1 ± 0.01) for neurons from control mice (Figure 8E).

By the 10th day of cultivation, in Satb2-deficient neurons, and control neurons, Ca^2+^-signals are suppressed after GABA application, it occurs when low-amplitude signals are recorded in response to 300 µM of the agonist (Figure 8D, black and purple curve). At the same time, Ca^2+^-signals with an amplitude of 0.04 are recorded even in response to 0.3 µM of GABA applied to mature Satb2-null neurons, and a subsequent increase in the concentration of GABAR activator results in increased amplitude of Ca^2+^-responses (Figure 8D, red curve). The EC50 value for activation of GABA receptors of Satb2-deficient neurons is 171 ± 4.67 µm (*n* = 0.93 ± 0.12) (Figure 8F), which is higher even than that for control neurons suggesting a switch of the GABAergic system from excitatory to inhibitory. To activate GABAR Satb2-null neurons, the EC50 value should be 10.2 ± 1.3 µm, which is almost 3 times less as compared to control neurons (Figure 8F).

Therefore, Satb1 knockout affects the activity of the GABAergic system and the process of switching the system from excitatory to inhibitory resulting in a lower sensitivity of immature Satb1-deficient and Satb1-null cortical neurons to GABAR-GABA, the natural agonist, while high amplitudes of Ca^2+^-signals after application of this agonist are typical for neurons from control mice (Satb1^+/+^ * Nex^Cre/+^). However, during the maturation of control neurons in the tissue sample and the development of the neural network, Ca^2+^-signals of the cells from control mice are almost completely suppressed after GABA application suggesting a switch of the GABAergic system to inhibitory. Oppositely, it is shown that Ca^2+^-signals are generated by Satb1-deficient and Satb1-null neurons after application of even micromolar GABA concentrations and the EC50 value decreases for GABA receptors.

However, as shown by vitality assays, in cell cultures obtained from mice with a deletion of Satb1, there was no massive apoptotic or necrotic cell death, compared with the control (Satb1^+/+^ * Nex^Cre/+^) (Supplementary, Figure S8). Therefore, on 3 DIVs in the control, necrotic cell death was detected only in single cells (Supplementary, Figure S8A, top line—PI; Figure S7C) and averaged 3 ± 2% (Supplementary, Figure S8E). At the time, in the control group, there were 17 ± 6% of cells in the early stage of apoptosis, which might be associated with the utilization of cells damaged during isolation from the cerebral cortex tissue (Supplementary, Figure S8C,E); and in the late stages of apoptosis, no more than 1% cells. In the Satb1-deficient group, there was no significant increase in the number of both necrotic cells (6 ± 4%) and cells in the early (20 ± 7%) and late (1%) stages of apoptosis (Supplementary, Figure S8A, middle line—PI; Figure S7C,E), compared to controls. However, in the Satb1-null group on the third day of cultivation, a significant (*** *p* ≤ 0.0001) increase in the number of necrotic cells was revealed—16 ± 5% (Supplementary, Figure S8A, bottom line—PI; Figure S7C,E), while differences in the induction of apoptosis are not significant in comparison with the control. In general, in the process of culturing cells from three experimental groups, a trend towards a decrease in the number of necrotic and apoptotic cells was observed by 10 DIV (Supplementary, Figure S8B,D,E). Only for control cultures, there was a significant increase in the number of cells at the late stage of apoptosis 16.7 ± 7% (Supplementary, Figure S8D,E), and the number of necrotic cells (Supplementary, Figure S8B, top line—PI) and cells at the early stages of apoptosis did not change significantly compared to 3 DIV.

In the case of deletion of Satb2, no significant differences were found in the number of cells dying by one pathway or another by 3 DIV (Supplementary, Figure S9A,C,E). Interestingly, culturing cells with a Satb2 deletion up to 10 DIV leads, as in the case of Satb1, to a decrease in the number of necrosis and cells at a late stage of apoptosis (Supplementary, Figure S9B,D,E).

As for Satb2-deficient neurons, it was found that during maturation of these neurons, Ca^2+^-signals are suppressed after GABA application; Ca^2+^-dynamics and the EC50 value of these neurons are similar to the cortical neurons from control mice. However, Satb2-null neurons demonstrate higher sensitivity to GABAR activation at the early stage of the neural network development, and the same dynamics is also seen in the late stages of cultivation when neurons produce Ca^2+^-signals with high amplitudes in response to 300 nM GABA, and the EC50 value reduces by less than two times as compared to that in early stages of cultivation.

## 4. Discussion

Satb1 is a risk factor for the development of neurodegenerative diseases, particularly Parkinson’s disease (PD) [45,46]. In addition, it was shown that genetic ablation of SATB1 induces a senescence phenotype in human embryonic stem cell-derived DA neurons and contributes to the development of PD [47]. Satb2 is required for the development of a spinal exteroceptive microcircuit that modulates limb position [48]. As deletion in the transcription factor Satb2 leads to the development of epilepsy and developmental delay in people [19,49] and duplication of Satb2 can also induce epilepsy and autistic behavior [50], the importance of investigation of the molecular and cellular mechanisms of the Satb2 effect in the brain leading to the induction of neurodegenerative processes is apparent. Glial cells, astrocytes, and microglia are also involved in the induction and pathogenesis of most neurodegenerative disorders [51]. It has been shown that the selective dysfunction or death of the neuronal population most at risk in each disease is not mediated solely by damage from the mutant protein within the target neurons [52]. In our article, Satb1- and Satb2-deletions were performed selectively in neurons, but, despite this, it is possible to assume disruptions in neuron-glial interactions [53] and requires more investigations.

Satb2 is abundantly expressed in pyramidal neurons in the cortex and hippocampus. Satb2 is involved in the regulation of brain development during embryogenesis [54,55] and has an effect on the learning process and memory formation in adult mice [56,57]. In this study, we describe the effects of Satb1 and Satb2 deletions on Ca^2+^-signals of cortical neurons when NMDAR and AMPAR are activated, and a smaller deletion effect is shown for KAR. In the cultured cortical neurons of Satb-deficient and Satb-null mice with deletions of these transcription factors, the amplitudes of Ca^2+^-signals are higher when NMDAR is activated due to increased expression of genes encoding the receptor subunits. Simultaneously, Ca^2+^-signals from neurons in response to the AMPAR agonist treatment are suppressed due to increased expression of the GluA2-subunit responsible for the formation of AMPAR that cannot conduct Ca^2+^ ions. Here, we report that Satb1 and Satb2 are involved in the mechanisms of NMDA receptor neurotransmission. There is evidence that Satb1 and Satb2 can affect intracellular signaling of neurons via chromatin looping of the NMDA receptor locus GRIN2B46 [58] and two schizophrenia-risk genes encoding the GABA synthesis enzyme (GAD1) and the calcium channel alpha subunit CACNA1C in the activation of cognitive and physical disturbances [59,60]. Furthermore, Satb1 and Satb2, a known chromatin structure organizer, contribute to the formation of synaptic spikes, participating in neuronal plasticity [44]. Interestingly, at lower mRNA levels of Grin2a and Grin2b in the cerebral cortex of Satb1-null and Satb2-null mice, as compared to Satb-deficient mice, a significantly higher level of the protein of GluN2A- and GluN2B- subunits were revealed and, as a result, increased amplitudes Ca^2+^-signals for NMDAR activation. This can be explained by the increased expression of the *Grin1* gene in the cortex of Satb-null mice, which encodes the GluN1-subunit, which is necessary for the transport of GluN2- subunits to the cell membrane [61]. It should also be noted that post-translational modification of proteins can significantly determine the amplitude of Ca^2+^-signals to agonists, especially in the case of AMPAR. If AMPAR lacks the GluA2-subunit, then the channel of this GluA2-lacking AMPA-receptor begins to conduct Ca^2+^ ions, and this is determined by post-translational modification of the GluA2 mRNA, in which the uncharged amino acid glutamine is replaced by a positively charged arginine in the receptor channel [62,63]. Moreover, the formation of GluA2-lacking AMPA-receptors can also occur in the presence of mRNA of GluA2 [64]. In our experiments, the level of expression of the *Gria2* gene was significantly lower in the neurons from Satb-null mice than from Satb-deficient mice. At the same time, the number of GluA2-subunits on the membrane of Satb-null neurons was significantly higher, as well as the degree of suppression of Ca^2+^-signals to the AMPAR activator application. This effect can be explained precisely by the differences in the activity of post-translational modification of GluA2-subunits in Satb-null and Satb-deficient neurons, although this requires additional experiments.

Restriction of [Ca^2+^]_i_ in the cortical neurons from mice with Satb1 and Satb2 deletions may have an antiepileptic effect and protect neuronal networks. It was found that PTZ (pentylenetetrazol) injections cause epileptiform seizures by activating the mamillo-thalamic tract and creating an imbalance between excitation and inhibition in the hippocampal neuronal network [65]. The Satb2 deletions reduce the neuronal excitability and excitatory inputs in pyramidal neurons of CA1, which contributes to the suppression of epilepsy when PTZ is administered [66]. CaMKII was found to be activated (at T286 on CaMKIIα subunits or T287 on CaMKIIβ subunits) in response to the input of Ca^2+^ ions through NMDAR [67,68] and T286A mutation that prevents the autophosphorylation and activation of CaMKIIα in mice significantly impairs NMDAR-dependent LTP in the hippocampal CA1 area and memory performance in a Morris water maze task [69]. Moreover, it is shown that overexpressing a GluN2B carboxyl-terminal fragment (839-1482aa) that disrupts the physiological interaction between NMDAR/CaMKII leads to severe deficits in hippocampal LTP and spatial learning in transgenic mice [69], which is not observed in our mice with the knockout of Satb1 and Satb2. For example, a mutation of the transcription factor Sip1 causes CaMKII expression impairment, and the phenomenon of hypoxic preconditioning disappears in cortical neurons [26], but the Satb1 and Satb2 knockout probably do not affect this important kinase.

Interestingly, the increased sensitivity of neurons of the cerebral cortex obtained from Satb-deficient and Satb-null mice to activation of NMDA receptors clearly correlates with an increased level of NMDAR subunits gene expression *Dlg3* gene (protein PSD-95). PSD-95 is not only a marker of neuronal maturity [70] and its level should increase during network development, but this protein is also involved in the regulation of NMDAR activity. It has been shown that PSD-95 is associated with the activation of the GluN2B-PSD95-nNOS signaling pathway [71]. The inhibition of any of the proteins of this pathway suppresses NMDAR-mediated NO production and protects neurons from excitotoxicity [72,73].

These changes in Ca^2+^-signaling and expression of receptor subunits may promote induction of epilepsy, neurodegenerative diseases, and in the case of sharp load on cells, they may lead to the activation of apoptosis. Moreover, it has been shown recently that Ca^2+^- signaling is also involved in brain swelling, TBI, and edema [74]. The research on the mechanisms of action of Satb1 and Satb2 transcription factors on the nervous system has been undertaken in the last decade and especially due to the development of Ca^2+^-signaling, so many results are incomplete. However, there is some evidence of changes in the expression of genes involved in Ca^2+^-signaling during Satb2 deletion. In neurons from mice with the Satb2 deletion [56], it is shown that the expression level of several genes associated with the maintenance of Ca^2+^-homeostasis (e.g., Cd36 [75], Plcz1 [76], Calb1 [77], Ramp3) changes [78].

Deletions in Satb1 and Satb2 lead to a decrease in the amplitude of Ca^2+^-responses to domoic acid (a KAR agonist) and a decrease in the expression of genes encoding KAR. These changes may be considered as a compensatory mechanism. It was shown that activation of neuronal networks by kainate does not correlate with Satb2 expression. Expression of Satb2 protein in the hippocampus is suppressed after neuronal hyperactivation initiated by glutamate, but in the case of intra-amygdala KA injection, no correlation between neuronal hyperexcitation and Satb2 expression was revealed [66,79]. Mice with Satb1 knockout are marked by anti-anxiety behavior in the open field test [57]. At the same time, a change in the expression of genes encoding CaMKII, Psd-95, and PKC may respond to the suppression of Ca^2+^-signals for DA application in Satb1- and Satb2-null neurons (Supplementary, Figure S10). Some kinases can regulate the activity and the density of kainate receptors on the cell membrane of neurons. Satb1- and Satb2-null mice are characterized by increased expression of the gene encoding CaMKII (by 4.7 times and 6.4 times, respectively, compared to control) (Supplementary, Figure S10). It is known that phosphorylation of GluK5 subunits (*Grik1*-gene) increases surface expression and decreases synaptic content of KARs in cultured hippocampal neurons [80]. Moreover, the level of Psd-95 expression is 5.1 and 7.3 times higher in Satb1- and Satb2-null mice (Supplementary, Figure S10), which can also contribute to a decrease in the amplitude of DA-induced Ca^2+^-signals.

The expression level of all PKC isoforms is higher in cells from Satb2-null mice (Supplementary, Figure S10B). These differences may also contribute to the suppression of Ca^2+^-signals upon KAR activation. It is known that SNAP25 and GluK4 (*Grik1*-gene) form a complex with the trafficking proteins PICK1 and GRIP in postsynaptic terminals. This complex is regulated by PKC phosphorylation. Hence, the stimulation of PKC triggers long-term depression of KAR-mediated synaptic responses [81].

The expression of glutamate and GABA receptors changes during the development of the neuronal network. The maintenance of the excitation/inhibition balance is needed for normal neurotransmission. γ-aminobutyric acid (GABA, Sigma-Aldrich, Cat. #A2129) is an inhibitory neuromediator regulating multiple processes in the organism, from the muscle tone to emotional responses. It should be noted that GABA becomes an inhibitory mediator only in the mature brain, whereas in the developing brain, GABA has an excitatory effect in the form of changes in the permeability of the cell membrane of the neurons to Cl^−^-ions. In immature nerve cells, the concentration of chlorine ions is higher than in the surrounding medium, and stimulation of GABA receptors leads to a release of these anions out of the cell followed by membrane depolarization [82]. During brain development, glutamate, the main excitatory transmitter, matures, and GABA can function as an inhibitory (hyperpolarizing) neurotransmitter [83].

Mutations in a series of genes lead to impaired migration, maturation, or functioning of GABAergic neurons, thus contributing to the development of seizures [84]. In this work, we found that in neurons derived from the cortex of null mice with Satb1- and Satb2- deletions, no decrease in sensitivity of GABA receptors to agonists occurs during cultivation and maturation of the neural network. On the one hand, this effect may be explained by the high expression level of GABA(a) receptors at the late stages of cultivation of knockout neurons; and on the other hand, by the immaturity of the neural network. It is known that Satb1 and Satb2 are both involved in the regulation of brain development in the embryonic and early postnatal periods. Satb1 and Satb2 are expressed in the developing central nervous system, but their expression patterns do not overlap. In the developing brain, Satb1 is expressed in the MZ and piriform cortex, while Satb2 is expressed predominantly in superficial layers of the neocortex and subiculum [17]. In Satb1-null mice, the cortex develops without significant abnormalities. However, reduced density of dendritic spines playing a key role in synaptic transmission and plasticity was shown in pyramidal neurons in the postnatal period. Here, we demonstrate an increase in the expression of developmental markers during the cultivation of neurons with a deletion of Satb1 and Satb2. However, the level of expression of *Dcx* and *Syp* genes is lower in this case compared to control cells. These data may indicate a delay in the development of cell cultures with deletions of Satb1 and Satb2. In turn, other authors have shown that the expression of Doublecortin (*Dcx* gene), PSD-95 (*Dlg4* gene), and Synaptophysin (*Syp* gene) is enhanced during the development of neuronal cultures [85,86].

In addition, the transcription factor Satb1 regulates a number of early genes (*Fos, Fosb, Egr1, Egr2, Arc, Bdnf*) involved in synaptic plasticity and neuroprotection [31]. Thus, Satb1 is necessary to regulate the proper temporal dynamics of immediate early gene expression and facilitate neuronal plasticity in the cortex [44,48]. There is evidence that transcript levels of Satb2 are low (30% of Satb1 level) on the first day after the birth of mice, and then it slows down as the level of protein decreases [16]. Satb2 is involved in the differentiation of neurons [54], stem cells [87], and osteoblasts [88]. It was shown that Satb2 plays an important role in the postnatal period of development [30,54,56]. Satb2 directly binds to the BCL11B locus and recruits the Ski protein and induces the nucleosome remodeling deacetylase (NuRD) complex to initiate chromatin modifications inhibiting BCL11B expression [89]. Interestingly, the molecular mechanisms activated by Satb2 in the postnatal period differ completely from those in embryogenesis. It should be noted that these molecular and cellular mechanisms of Satb2 in the adult brain are still poorly studied. For instance, germline Satb2-deficient mice die perinatally [88], and in contrast to the layer-specific embryonic expression, adult CNS Satb2 is expressed in pyramidal neurons of all layers of the cerebral cortex and the hippocampal CA1 area [90]. Patients with SATB2 haploinsufficiency did not show abnormalities of the corpus callosum [91,92], as well as mice with Satb2 knockout [54]. In the case of Satb1-deletion, we observed an increase in the amplitudes of Ca^2+^-signals and a decrease in the EC50 value in response to GABA treatment during neural network maturation in the cortex of Satb1-deficient and Satb1-null mice. In the light of it, some evidence indicates that in interneurons of heterozygous mice with Satb1 knockout, there is a reduction of KCC2 expression (K^+^-Cl^−^-co-transporter-2). Since KCC2 is considered as a marker of interneuron maturation, a disruption of the maturation mechanisms of certain subtypes of cortical inhibitory interneurons of the cortex becomes possible [14]. In the immature brain, due to the reverse Cl^−^ gradient, the action of GABA as an excitatory neurotransmitter determines the migration and maturation of neurons, as well as the integration of neural networks [83]. Inhibition of GABA(A)-receptors with bicuculline and picrotoxin results in a lower percentage of GABAergic neurons expressing Satb1 in 48 h, which indicates that neuronal excitability and Ca^2+^-activity contribute to the regulation of Satb1 expression [14]. Spontaneous activity of neurons during the development of the CNS effects such processes of development as cell migration, synaptogenesis, and neuronal maturation [93]. Satb1 expression is weakly pronounced 24 h after seeding in GABAergic neurons derived from dorsal telencephalon of mouse embryos, but the rate of Satb1^+^—GABAergic neurons rises during cultivation. In this case, a depolarizing stimulus induced by the application of 40 mM KCl to these cultures leads to an increase in [Ca^2+^]_i_ in neurons through L-type voltage-gated Ca^2+^-channels [94] and is accompanied by a significant rise of the Satb1 expression rate. This depolarization effect on Satb1 expression was blocked by the blocker of Ca^2+^-channels of L-type—nifedipine [14]. Deletion of Satb1 in SST^+^-interneurons results in abnormal integration of these interneurons in the process of network development, which can lead to their damage and death [28]. In addition, selective deletion of Satb1 in inhibitory interneurons initiates the impaired inhibition mechanism of pyramidal neurons, which at the in vivo level is manifested in the induction of epileptiform activity in the cortical layers of the brain [95]. Therefore, Satb1 knockout causes not only the death of inhibitory interneurons in the early postnatal period but also contributes to the hyperexcitation of survived populations of interneurons. Increased excitability of SST^+^-interneurons deprived of Satb1 may be a homeostatic compensation that results from the reduced excitatory drive that cells with deletion receive or occur due to incomplete maturation of these neurons [28]. It is interesting that the obtained data can explain the induction of neurodegenerative processes upon deletion of Satb1/Satb2 transcription factors. At the same time, in future research, the use of high-throughput screening and computer-aided drug design [96,97] can provide a novel insight that can support the findings from this work.

## 5. Conclusions

Thus, we have demonstrated that deletion in the transcription factors Satb1 and Satb2 in cortical neurons leads to the change in glutamatergic and GABAergic neurotransmission resulting in a variation of the expression of genes encoding subunits of key glutamate receptors and their subunits, and GABA(A) receptor, as evidenced by a shift in the EC50 value for activation of these receptors.

## Figures and Tables

**Table 1:** Primer sequences for real-time polymerase chain reaction (RT-PCR).

Gapdh	Forward 5′-ccacggcaagttcaacggcac-3′ Reverse 5′-gatgatgacccttttggccccacc-3′
Grik1	Forward 5′-ggaggatgaggcggggacc-3′ Reverse 5′-gcatgctcttcgggaggcttcaaaac-3′
Grik2	Forward 5′-ggatgggaaatatggagcccaggatgat-3′ Reverse 5′-tcaggggagagaggattcaggaaggag-3′
Grin1	Forward 5′-tgacggtgagatggaagagctg-3′ Reverse 5′-ctgccatgttgtcgatgtccag-3′
Grin2a	Forward 5′- cagtaaaccaggccaataagcga-3′ Reverse 5′- atctgtatggcgttgggcttgt-3′
Grin2b	Forward 5′-gtcctccaaagacacgagcac-3′ Reverse 5′-gccctcctccctctcaatagc-3′
Gria1	Forward 5′-aggggaatgtggaagcaaggac-3′ Reverse 5′-ccaatcccagccctccaatcag-3′
Gria2	Forward 5′-agccaaggactcgggaagtaagg-3′ Reverse 5′-caccagcattgccaaaccaagg-3′
Gabra1	Forward 5′- tatctttgggcctggaccctcattctg-3′ Reverse 5′-ccataaggttgtttagccggagcactg-3′
Dcx	Forward 5′- gcaatggggaccgttacttcaa-3′ Reverse 5′- agccagcaacgcatcaaaactac-3′
Syp	Forward 5′- aggtgctgcagtgggtctttg-3′ Reverse 5′- actctccgtcttgttggcacact-3′
Dlg3	Forward 5′- aggagatcacattggaaaggggtaa-3′ Reverse 5′- tggtgataaagatggatgggtcgt-3′
Pik3ca	Forward 5′-ctgagatgggagctgggactgc-3′ Reverse 5′-gtgtccacgtgttagacagaacactg-3′
Pik3cb	Forward 5′-gaggttatgagtgtgcttccgccctat-3′ Reverse 5′-agtcttcgtgtttcgtcttccagttcctc-3′
Pik3cg	Forward 5′-gctgcggagttctaccaccgattg-3′ Reverse 5′-caggtagtctgggagaggtttggacg-3′
Camk2a	Forward 5′-gagcagcaggcatggtttgggt-3′ Reverse 5′-ggtgcttgagagcctcagcgg-3′
Prkca	Forward 5′-ccaacgactccacggcgtctc-3′ Reverse 5′-tgcttgtgaacattcatgtcgcaggtgt-3′
Prkce	Forward 5′-tgatcatcgatctctcgggatcatcggg-3′ Reverse 5′-gcccacctcgtcaggggtttc-3′
Prkcg	Forward 5′-tggttcaccgccgatgccac-3′ Reverse 5′-ccgcaaagggagggcacg-3′
Prkcg	Forward 5′-gccccccaacatggactgtctct-3′ Reverse 5′-gggttgtcagtgccacctgcgat-3′

**Table 2:** The effect of Satb1 and Satb2 deletion on agonist-induced Ca^2+^-responses of cortical neurons.

	EC50/ΔF(Fmax–Fmin)		
	NMDAR	AMPAR	KAR
**Control**	6.39 µM/0.139	0.004 µM/0.285	0.32 µM/0.181
Satb1^fl/+^ * Nex^Cre/+^	9.94 µM/0.153 *	0.018 µM/0.222 ***	0.39 µM/0.137 ^n/s^
Satb1^fl/fl^ * Nex^Cre/+^	1.26 µM/0.254 **	0.002 µM/0.171 ^n/s^	0.27 µM/0.239 ^n/s^
Satb2^fl/+^ * Nex^Cre/+^	6.19 µM/0.173 *	0.025 µM/0.221 ***	0.176 µM/0.179 ***
Satb2^fl/fl^ * Nex^Cre/+^	2.83 µM/0.184 ***	0.092 µM/0.222 ***	0.49 µM/0.126 ***

Statistical analyses were performed by paired t-test. Significance between groups means * *p* < 0.05, ** *p* < 0.01, and *** *p* < 0.001 comparing Satb-deficient group and Satb-null group with control mice; n/s—data not significant.

## Data Availability

The data presented in this study are available on request from the corresponding author.

## Supplementary Materials

The following are available online at https://www.mdpi.com/article/10.3390/ijms22115968/s1, Figure S1: Immunocytochemical staining of control cell culture (Satb2^+/+^ * Nex^Cre/+^) and cultures obtained from deficient (Satb1^fl/+^ * Nex^Cre/+^) and null (Satb1^fl/fl^ * Nex^Cre/+^) mice with deletion of Satb1 or Satb2 with astrocytic marker, antibodies against glial fibrillary acidic protein (GFAP, green). Figure S2: Ca^2+^-responses of neurons obtained from the cerebral cortex of control mice. Figure S3: Identification of neurons expressing Satb1 and Satb2 in cell cultures of the cerebral cortex of control mice (Satb1^+/+^ * Nex^Cre/+^ and Satb2^+/+^ * Nex^Cre/+^) using immunocytochemical staining on day 10 in vitro. Figure S4: The role of activity of the Ca^2+^-permeable AMPA receptors in generation of the Ca^2+^-signals on 10 μM FW applications in the cortical neurons derived from the Satb1-null (green), Satb2-null (red) and Control (black) mice. Figure S5: The effect of deletion in the transcription factors Satb1 and Satb2 on the Ca^2+^-signals produced by cortical neurons after activation of NMDARs (A,B), AMPARs (C,D) and KARs (E,F) in the presence of selective receptor antagonists. Figure S6: Immunocytochemical staining of control cell culture (Control, Satb1^+/+^ * Nex^Cre/+^) and cultures obtained from Satb1-deficient (Satb1^fl/+^ * Nex^Cre/+^)- and Satb1-null (Satb1^fl/fl^ * Nex^Cre/+^) mice with neuronal marker (NeuN), inhibitory synaptic marker, vesicular GABA transporter (VGAT) and their merge at early (3 DIV, A) and late (10 DIV, B) times of cell cultivation. Figure S7: Immunocytochemical staining of control cell culture (Control, Satb2^+/+^ * Nex^Cre/+^) and cultures obtained from Satb2-deficient (Satb2^fl/+^ * Nex^Cre/+^)- and Satb2-null (Satb1^fl/fl^ * Nex^Cre/+^) mice with neuronal marker (NeuN), inhibitory synaptic marker, vesicular GABA transporter (VGAT) and their merge at early (3 DIV, A) and late (10 DIV, B) times of cell cultivation. Figure S8: Survival of cortical cells obtained from control (Satb1^+/+^ * Nex^Cre/+^), deficient (Satb1^fl/+^* Nex^Cre/+^) and null (Satb1^fl/fl^ * Nex^Cre/+^) mice during in vitro cultivation. Figure S9: Survival of cortical cells obtained from control (Satb2^+/+^ * Nex^Cre/+^), deficient (Satb2^fl/+^ * Nex^Cre/+^) and null (Satb2^fl/fl^ * Nex^Cre/+^) mice during in vitro cultivation. Figure S10: Deletion in Satb1 and Satb2 affect the expression of genes, encoding kinases produced by the cortical cell cultures. Figure S11: Ca^2+^ -responses of neurons obtained from the cerebral cortex of Satb1-null.
