# Supplementary material for: Role of Satb1 and Satb2 Transcription Factors in the Glutamate Receptors Expression and Ca2+ Signaling in the Cortical Neurons In Vitro
Source: Int J Mol Sci. 2021 May 31;22(11):5968. doi: 10.3390/ijms22115968 (PMC8198236; doi:10.3390/ijms22115968)
Supplement: Supplementary file 1 [file ijms-22-05968-s001.zip › ijms-1237760-SI.pdf]

Supplementary

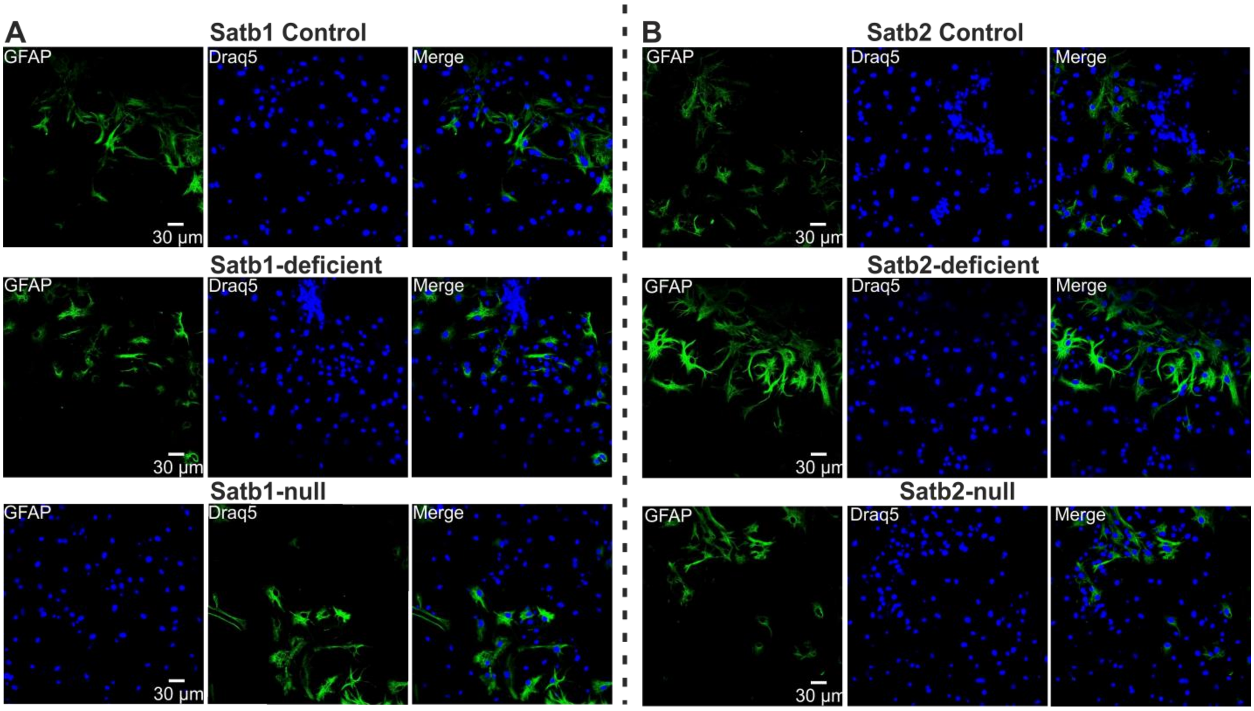

Supplementary. Figure S1. Immunocytochemical staining of control cell culture (Satb2$^{+/+}$ * Nex$^{Cre/+}$) and cultures obtained from deficient (Satb1$^{fl/+}$ * Nex$^{Cre/+}$) and null (Satb1$^{fl/fl}$ * Nex$^{Cre/+}$) mice with deletion of Satb1 or Satb2 with astrocytic marker, antibodies against glial fibrillary acidic protein (GFAP, green). The nuclei of all cells stained with Draq5 are shown in blue. Age of cortical cell cultures 10 DIV.

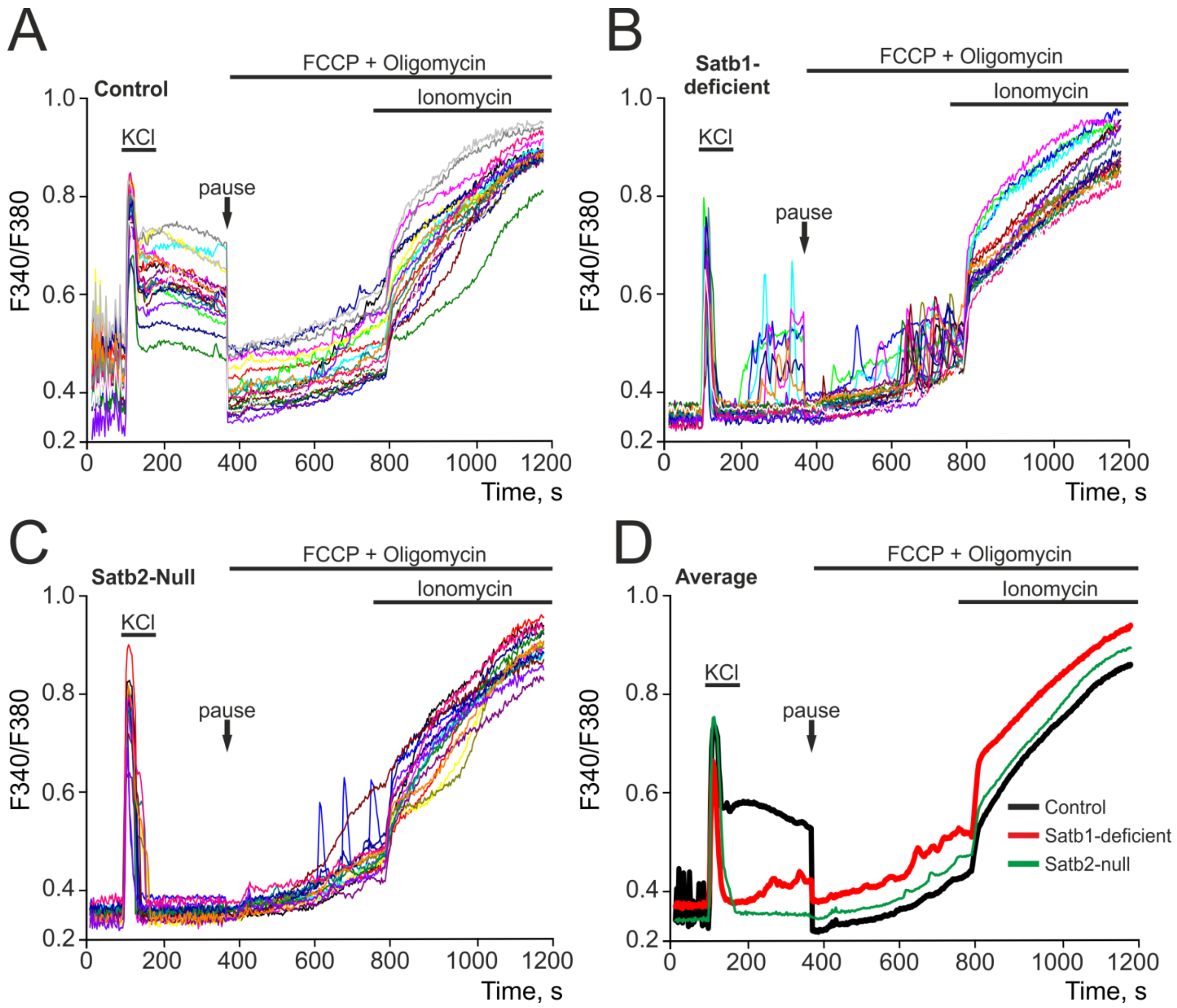

Supplementary. Figure S2. Ca^{2+}-responses of neurons obtained from the cerebral cortex of control mice (A), Satb1-deficient (B) and Satb2-null (C) mices to the application of Ca^{2+}-ionophore, Ionomycin (1 μM) with the mitochondrial uncoupler FCCP (1 μM) and an inhibitor of ATP-synthase (Oligomycin 2μg / ml). D – Averaged Ca^{2+}-signals from several tens of neurons, which show insignificant differences in the maximum Ca2+-responses between the control and experimental groups (Satb1-deficient and Satb2-null). KCl – short-term application of 35 mM KCl. Cortical cultures 10DIV.

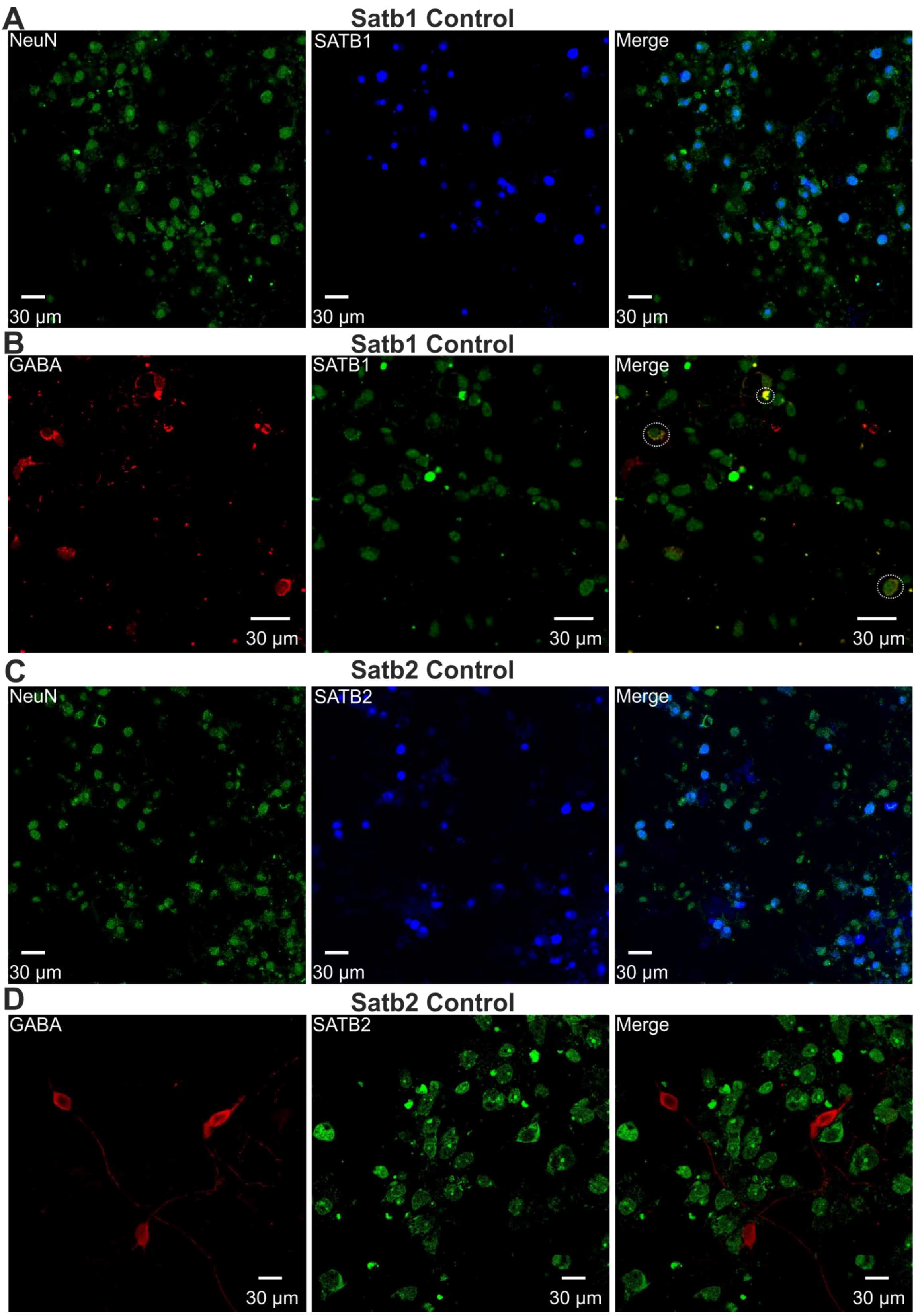

Supplementary. Figure S3. Identification of neurons expressing Satb1 and Satb2 in cell cultures of the cerebral cortex of control mice (Satb1$^{+/+}$ * Nex$^{Cre/+}$ and Satb2$^{+/+}$ * Nex$^{Cre/+}$) using immunocytochemical staining on day 10 *in vitro*. A, C – Images of cell culturestained with antibodies against neuronal marker (NeuN) and transcription factors Satb1 (**A**) or Satb2 (**C**). **B, D** – Images of cell cultures stained with antibodies against the

GABAergic neuronal marker – GAD65/67 (GABA), and transcription factors Satb1 (**B**) or Satb2 (**D**). Panel **D** – GABAergic neurons with anti-Satb1 antibody fluorescence are marked with white circles.

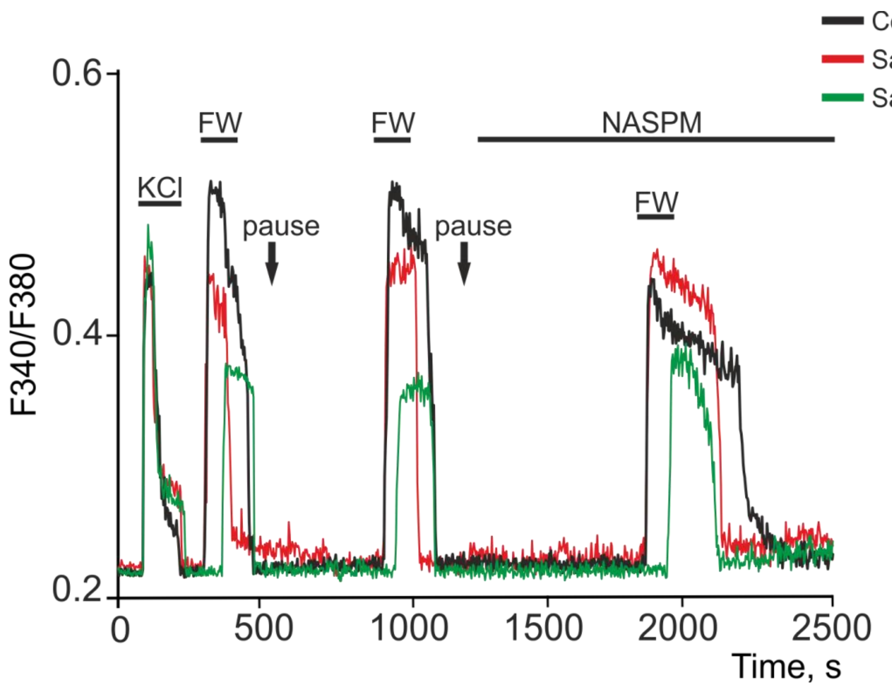

Supplementary. Figure S4. Fig. 2. The role of activity of the Ca^{2+}-permeable AMPA receptors in generation of the Ca^{2+}-signals on 10 μM FW applications in the cortical neurons derived from the Satb1-null (green), Satb2-null (red) and Control (black) mice. Average Ca^{2+}-responses of the cortical neurons to the FW applicationafter30minincubationwith100μM an antagonist of the calcium-conductive AMPA receptors (1-Naphthyl acetyl spermine, NASPM). Pause – marked 10-minute periods when the recording of Ca^{2+}-dynamics was not performed. KCl – short-term application of 35 mM KCl. Cortical cultures 10DIV.

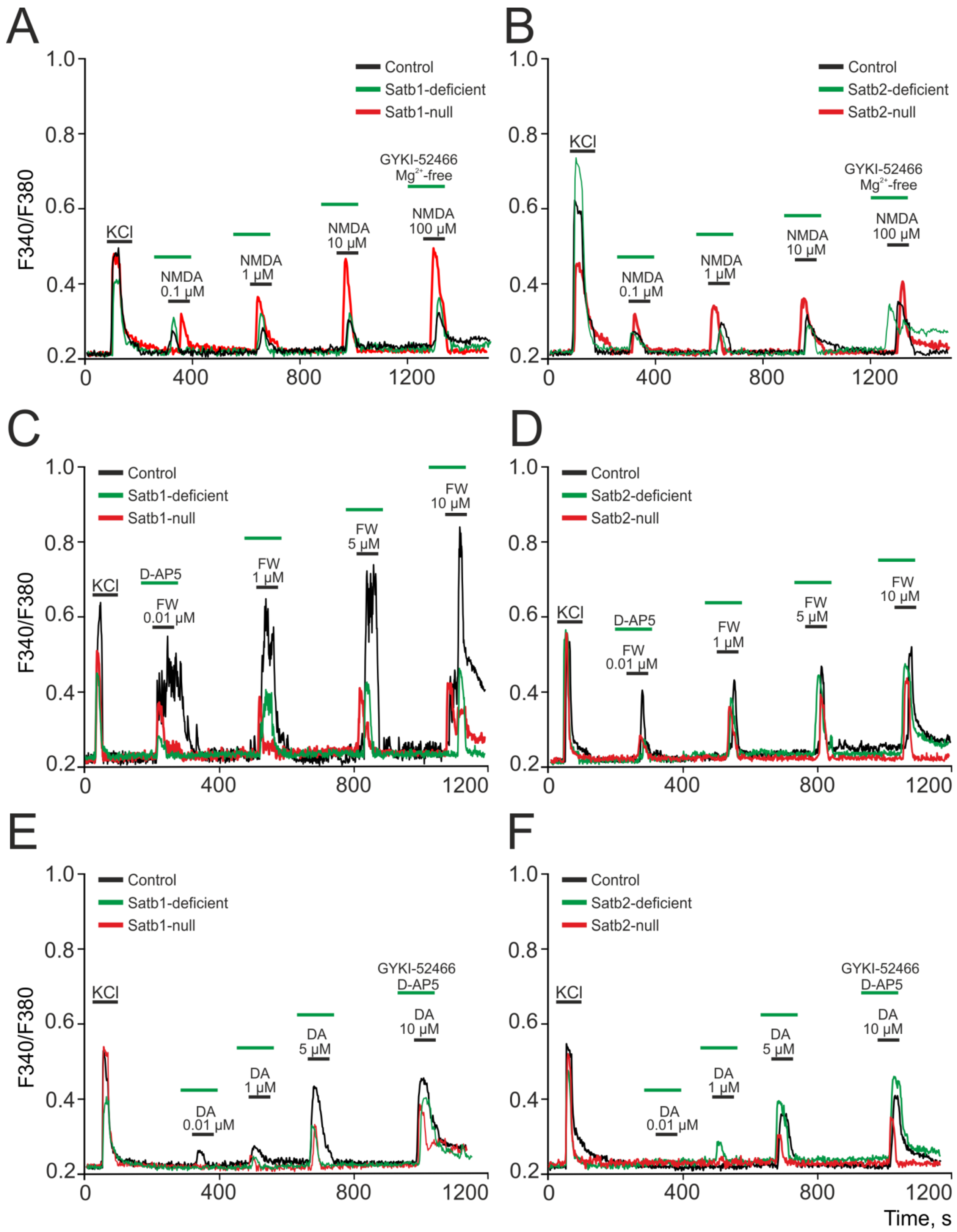

Supplementary. Figure S5. The effect of deletion in the transcription factors Satb1 and Satb2 on the Ca^{2+}-signals produced by cortical neurons after activation of NMDARs (A, B), AMPARs (C, D) and KARs (E, F) in the presence of selective receptor antagonists. A, B - Application of NMDA in Mg^{2+}-free medium with 30 µM an selective antagonist of AMPARs, GYKI-52466 to the neurons of brain cortex isolated from the Satb1- (**A**) and

Satb2- knockout mice (**B**). **C, D -** Application of FW with 10 μM an selective antagonist of NMDARs, D-AP5 to the neurons of brain cortex isolated from the Satb1- (**C**) and Satb2- knockout mice (**D**). **E, F -** Application of Domoic acid (DA) with 10 μM an selective antagonist of NMDARs, D-AP5 and 30 μM an selective antagonist of AMPARs, GYKI-52466 to the neurons of brain cortex isolated from the Satb1- (**E**) and Satb2- knockout mice (**F**). Averaged Ca^{2+}-responses for neurons from individual experiments are represented.

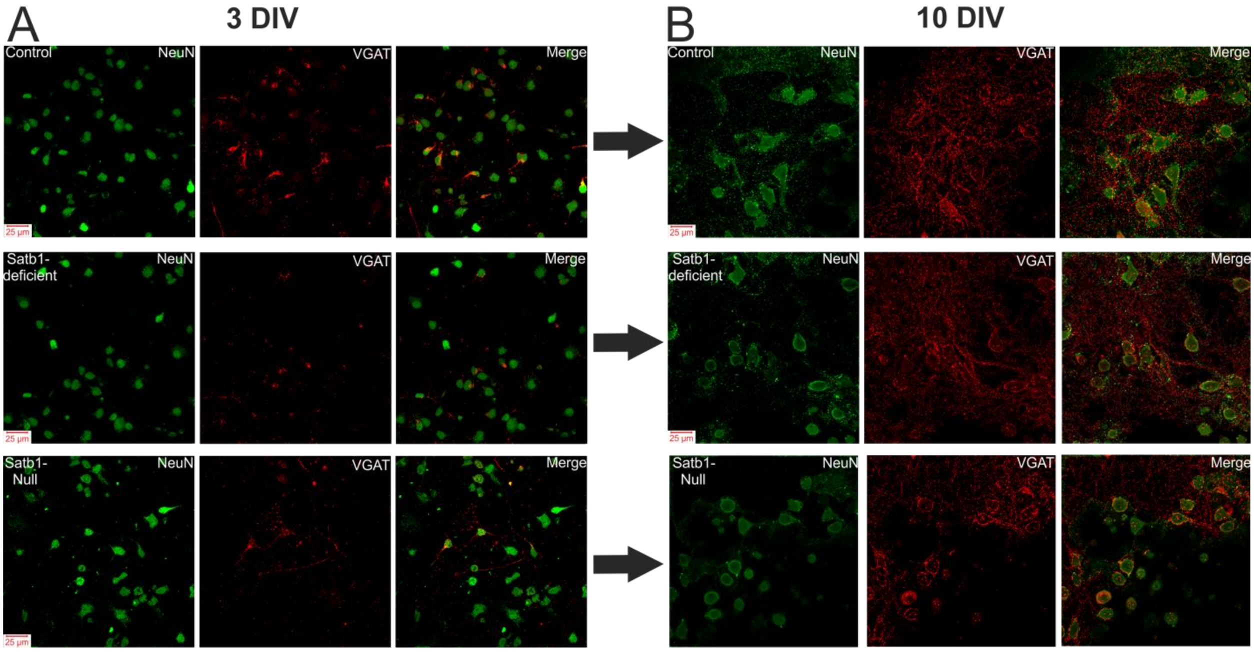

Supplementary. Figure S6. Immunocytochemical staining of control cell culture (Control, Satb1$^{+/+}$ * Nex$^{Cre/+}$) and cultures obtained from Satb1-deficient (Satb1$^{fl/+}$ * Nex$^{Cre/+}$)- and Satb1-null (Satb1$^{fl/fl}$ * Nex$^{Cre/+}$) mice with neuronal marker (NeuN), inhibitory synaptic marker, vesicular GABA transporter (VGAT) and their merge at early (3 DIV, A) and late (10 DIV, B) times of cell cultivation.

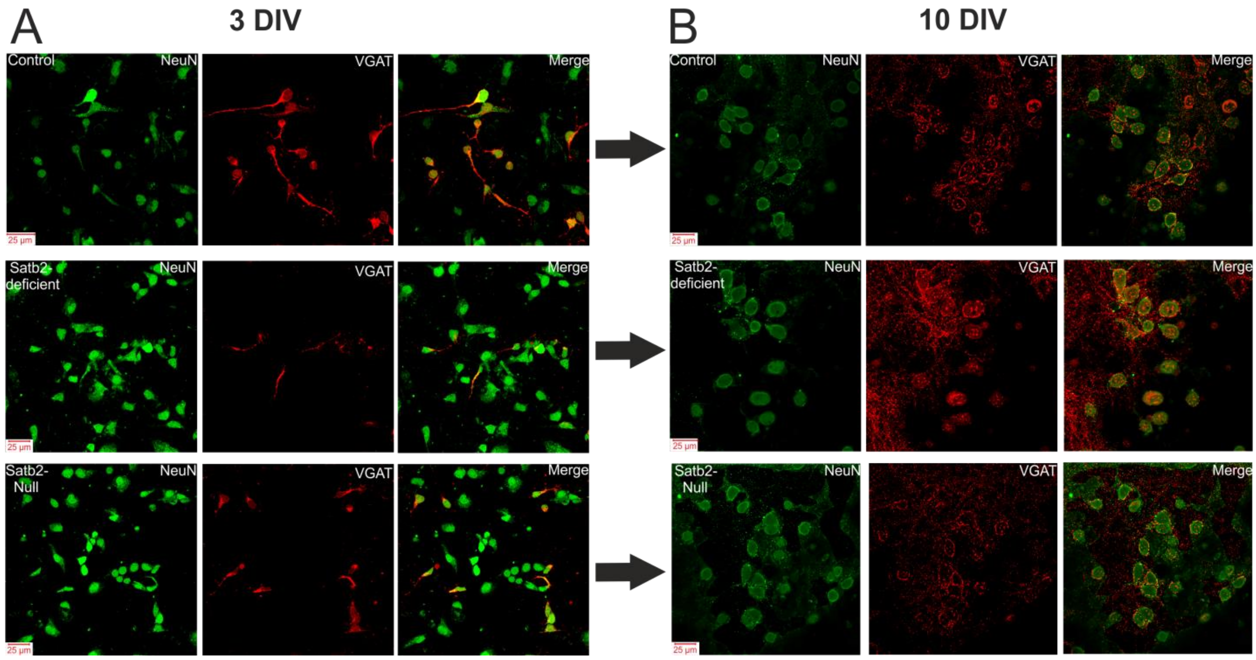

Supplementary. Figure S7. Immunocytochemical staining of control cell culture (Control, $Satb2^{+/+}$ * $Nex^{Cre/+}$) and cultures obtained from Satb2-deficient ($Satb2^{fl/+}$ * $Nex^{Cre/+}$)- and Satb2-null ($Satb1^{fl/fl}$ * $Nex^{Cre/+}$) mice with neuronal marker (NeuN), inhibitory synaptic marker, vesicular GABA transporter (VGAT) and their merge at early (3 DIV, A) and late (10 DIV, B) times of cell cultivation.

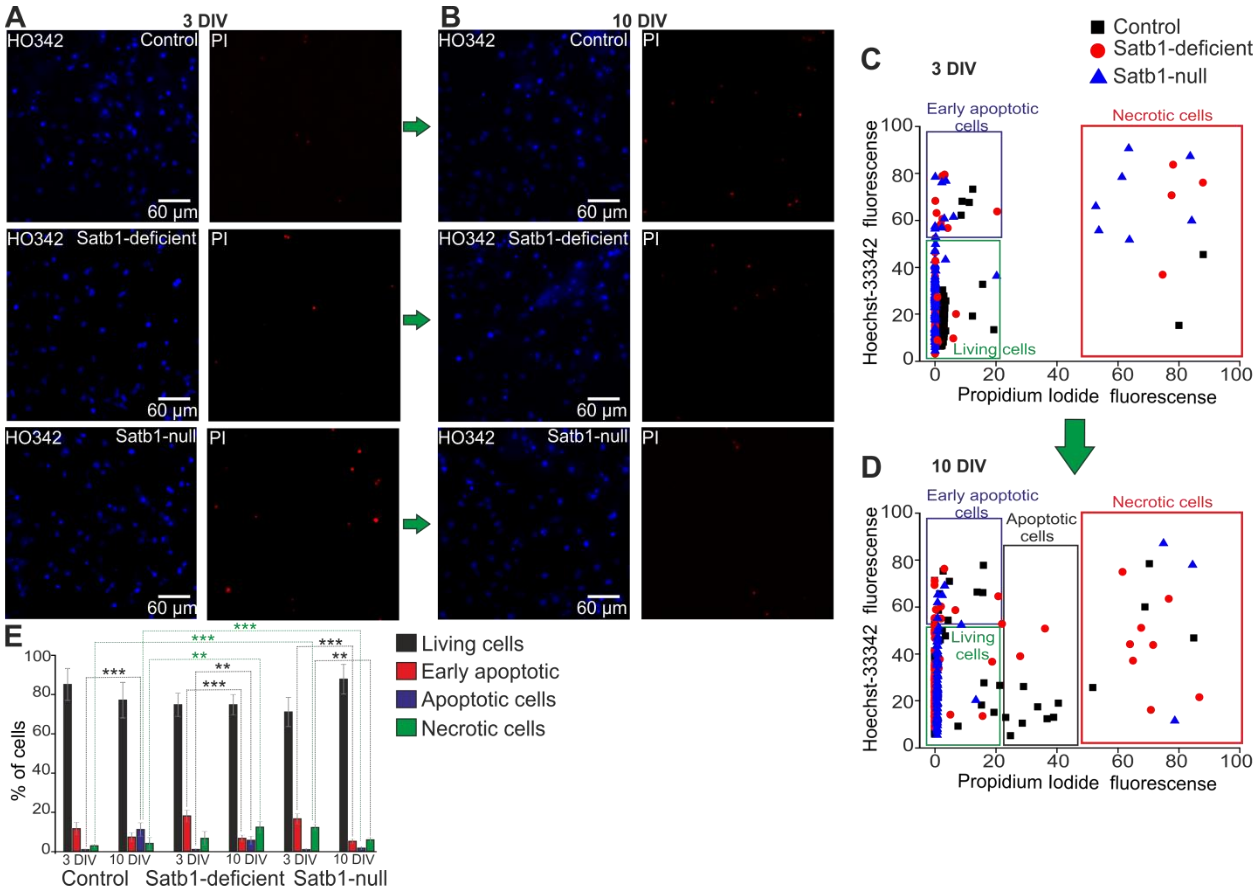

Supplementary. Figure S8. Survival of cortical cells obtained from control (Satb1$^{+/+*}$ Nex$^{Cre/+}$), deficient (Satb1$^{fl/+*}$ Nex$^{Cre/+}$) and null (Satb1$^{fl/fl*}$ Nex$^{Cre/+}$) mice during *in vitro* cultivation. A, **B** – Double staining of cortical cells with Hoechst 33342 (HO343) and propidium iodide (PI) on the 3 DIV (**A**) and on the 10 DIV (**B**). **C**, **D** – Cytogram demonstrating the viability of cells in different experimental groups on the 3 DIV (**C**) and on the 10 DIV (**D**). Y-axis – Hoechst 33342 fluorescence; X-axis – PI fluorescence. **E** – The histogram shows in percentage the portion of living cells (black column) and cells in which the processes of early apoptosis (red column), apoptosis (violet column) and destruction (necrosis, green column) were observed.

Statistical analyses were performed by paired t-test. Differences in the number of surviving cells are not significant when comparing the experimental Satb1-deficient and Satb1-null groups with the control for both 3 DIV and 10 DIV. The differences between the individual survival parameters studied between the experimental groups are marked with asterisks – *P<0.05, **P<0.01, and ***P<0.001. Experiments was repeated three times (n = 3) using cultures from different passages.

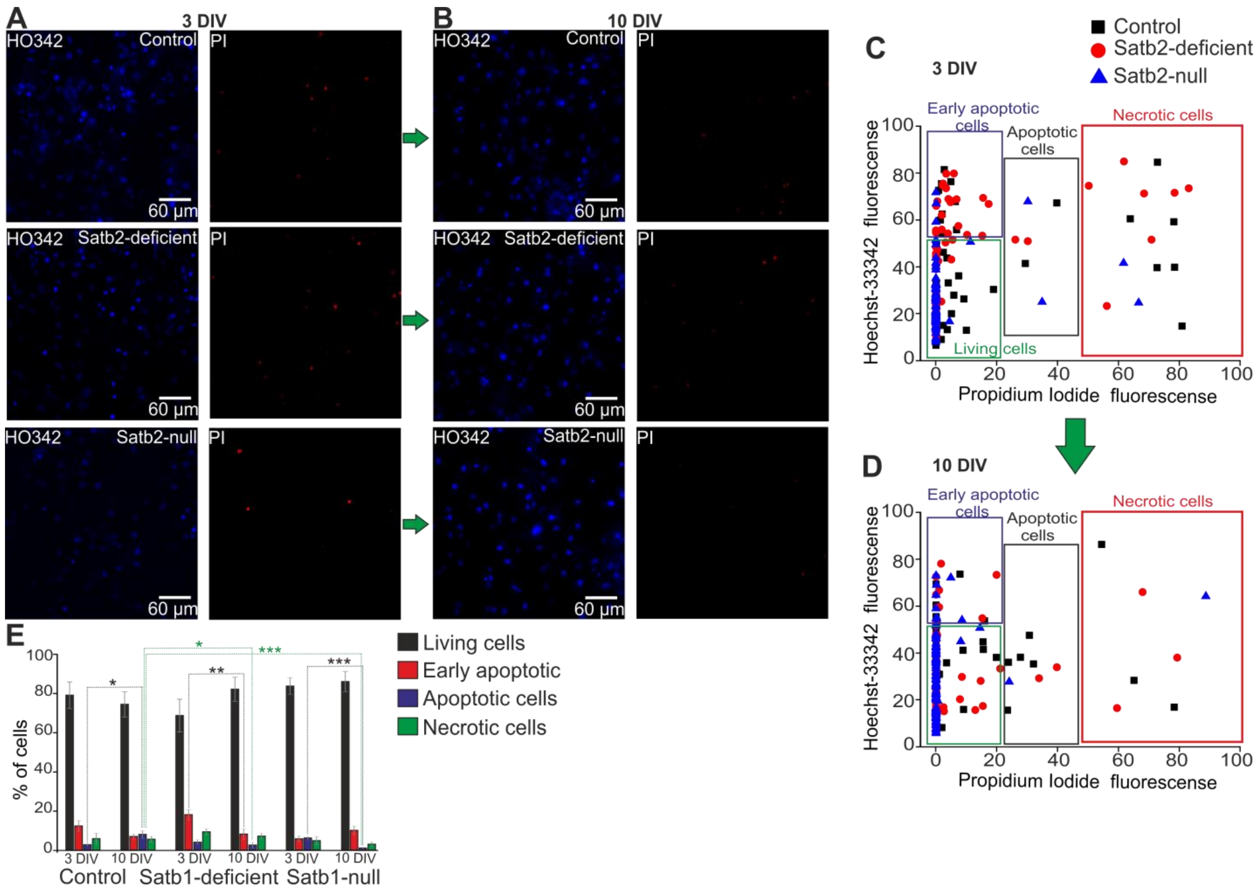

Supplementary. Figure S9. Survival of cortical cells obtained from control (Satb2$^{+/+*}$ Nex$^{Cre/+}$), deficient (Satb2$^{fl/+*}$ Nex$^{Cre/+}$) and null (Satb2$^{fl/fl*}$ Nex$^{Cre/+}$) mice during *in vitro* cultivation. A, B – Double staining of cortical cells with Hoechst 33342 (HO343) and propidium iodide (PI) on the 3 DIV (**A**) and on the 10 DIV (**B**). **C, D** – Cytogram demonstrating the viability of cells in different experimental groups on the 3 DIV (**C**) and on the 10 DIV (**D**). Y-axis – Hoechst 33342 fluorescence; X-axis – PI fluorescence. **E** – The histogram shows in percentage the portion of living cells (black column) and cells in which the processes of early apoptosis (red column), apoptosis (violet column) and destruction (necrosis, green column) were observed. Statistical analyses were performed by paired t-test. Differences in the number of surviving cells are not significant when comparing the experimental groups Satb2-deficient and Satb1-null with control for both 3 DIV and 10 DIV. The differences between the individual survival parameters studied between the experimental groups are marked with asterisks – *P<0.05, **P<0.01, and ***P<0.001. Experiments was repeated three times (n = 3) using cultures from different passages.

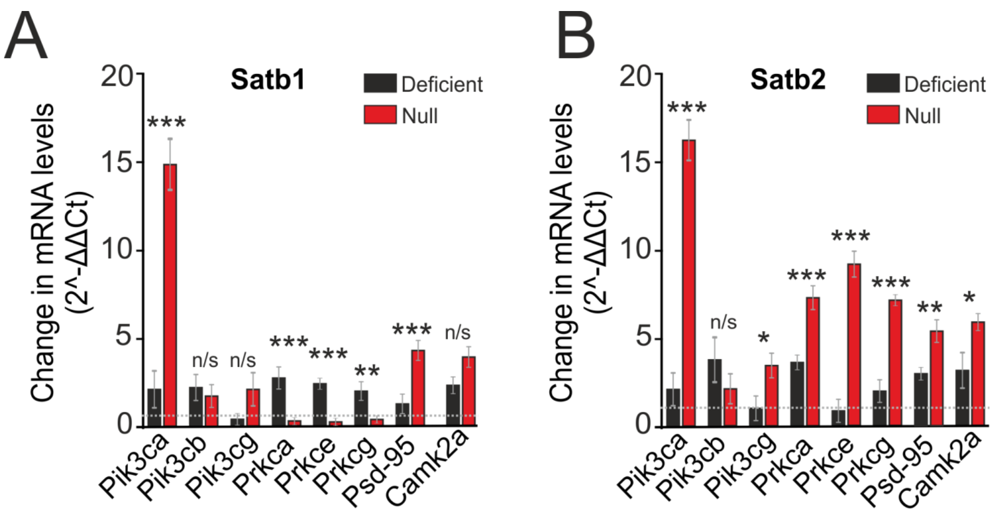

Supplementary. Figure S10. Deletion in Satb1 and Satb2 affect the expression of genes, encoding kinases produced by the cortical cell cultures. **A**, **B** – Alteration in the expression of genes, encoding isoforms of PI3K (Pik3ca, Pik3cb, Pik3cg), PKC (Prkca, Prkce, Prkcg), PSD-95 (Psd-95) and CaMKII (Camk2a) in the cortical neurons in Satb-deficient and Satb-null mice with deletions of transcription factors Satb1 (**A**) and Satb2 (**B**). Data obtained on four (n=4) different cell cultures are presented. All values are given as mean ± SEM. Gene expression level was normalized to reference gene Gapdh and was presented relating control (neurons from $Satb^{+/+}$ * $Nex^{Cre/+}$-mice), that was considered as 1 (dashed line). Total RNA was obtained from 10 DIV cultures. Statistical analyses were performed by paired t-test. Comparison of gene expression was performed between Satb-deficient and Satb-null mice. The data were significant: **** – p <0.0001.

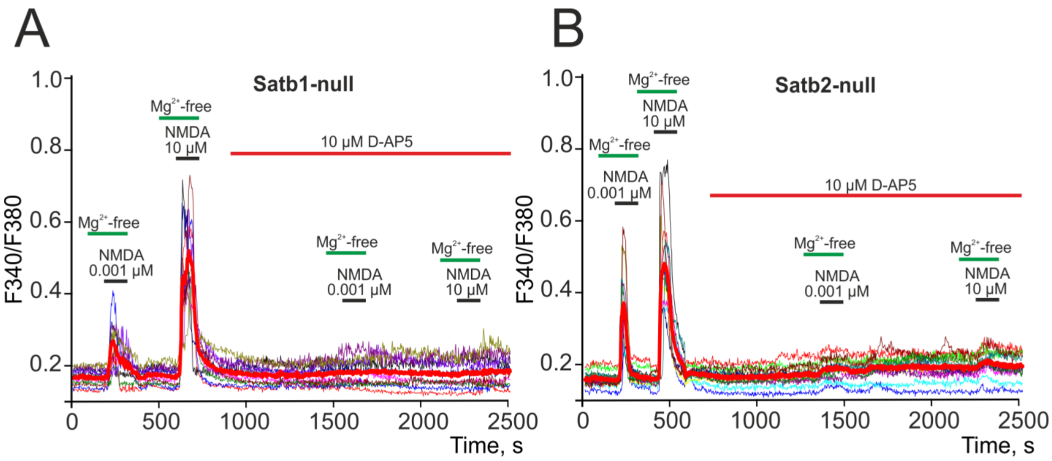

Supplementary. Figure S11. Ca^{2+} -responses of neurons obtained from the cerebral cortex of Satb1-null (A) and Satb2-null (B) mices to the application of NMDA in Mg^{2+}-free medium and after incubation with the selective NMDAR antagonist, D-AP5.Ca^{2+}-responses of neurons to NMDA are completely suppressed after incubation with a selective antagonist.
